# Supplementary material for: Trust, and distrust, of Ebola Treatment Centers: A case-study from Sierra Leone
Source: PLoS One. 2019 Dec 2;14(12):e0224511. doi: 10.1371/journal.pone.0224511 (PMC6886773; doi:10.1371/journal.pone.0224511)
Supplement: S4 File — Transcripts of six in-depth key-informant interviews. (DOCX) [file pone.0224511.s004.docx]

**S4a – Interview transcript Head nurse of the kindergarten**

Interviewer (I): Pleun Welmers

Interviewee (R): support staff

Translator (T): Musa

I: So, you worked as a nurse in the ETC. Do you remember when you started to work there at the kindergarten?

R: When I started to work there? Unfortunately, I do not remember now when I started to work there.

I: And do you remember was it in like November, December?

R: Well it was in November. Or October. Because I worked there for four months.

I: October. October, November, December and January.

R: No, then I think it was in November. November, December, January and February. In March the ETC ended. March.

I: OK, March. OK. And how did you get in contact to work at the ETC?

R: Well, I was at the Kenema Government Hospital during this crisis. [Staff] got infected at the government hospital. So, we were at home, discouraged, traumatized; we even did not know what to do since lots of us lost our relatives. Who [would take] care of us? Our husbands, our children, our parents. Lots of them, we lost there. Those people. All because of this sickness. So, we were at home very confused. [Then] this ET unit came at this time at Nyanyahun junction. So, we were there, and people got sick, and left their little children there, and they died with this Ebola. These children had nobody to take care of their Ebola. Maybe some children [would] die without knowing someone. So, the ETC decided to make a special place for kindergarten children, where the mother or father [had been] infected with Ebola. So, they brought them to the kindergarten unit. Because of the state of the Ebola, people were afraid, and of the nurses, because we are getting [the sickness], we were dying so much in the Kenema Government Hospital. So, we [were] afraid to take care of them. So, they decided to help the survivor nurses. Because they said when you have survived Ebola you will not [attack you] again and infect other people. So, they talk to the MS and the board of the KGH and they appoint[ed] us. They said those Red Cross people [will] come for you guys. They say they have opened an ET survivor unit for Ebola. So, they were having children of people that were getting affected. So, they wanted us as Ebola survivors, to take care of these Ebola children. Because we have already [been] infected and we are aware [of the disease]. So, if we are with them, we [can] take care, not like other nurses that are not infected. [These other nurses] will be afraid [of] them. They will not take proper care [of] them. They will judge them and get infected. So that is the reason why they implemented [this scheme, for] us. They hired us, and [deployed] us. When we were there to check the symptoms, then we called upon the people, [to] come and collect the child. That is how I got [employed?].

I: You were there when the first child came in the kindergarten?

R: If I can remember the first child?

I: No, you were there when the first child came to the kindergarten?

R: Yes, I started to work there, looking after these kindergarten children, those who had lost their parents. We were the first persons…, the first nurses. We were there working until the end of the ETC.

I: OK, and how many children were there in the kindergarten?

R: Yeah, I cannot remember now, because we had every day people that were infected. There was an example, we had a lot of infected people. So, all [the] children were taken to that house. They will check the kindergarten-children. For 21 days. They were quarantined for 21 days. To see if they [were] infected, so they can move there to the other infected people.

I: Ah, OK. Yeah, yeah. So, it is not… Did anybody stay longer then 21 days?

R: No, no. If you are there for 21 days and you don’t show any symptoms, you have to go back. But if you are there and you show any signs of symptoms, we take you to the ET unit. For example, we had two or three people there that got infected and died. We had an even younger one. I think we had even, I think the lady just gave birth to the child. So, the child was with us for a few days. The baby was…, even I was feeding him. But after three or four days he died.

I: Do you know [if] when they were in quarantine, they could give the infection to other children?

R: Well, you could not give it to the other ones. Because as soon as we see you, [you are] joking before, you are playing before, you are eating, [but] the day you become sad, we touch you and we see [if you have] the sign that you are infected. We inform the others at the ET Unit. That is how we do it, we take care and observe for 21 days.

I: How many nurses were there in the kindergarten?

R: [Counts] 1, 2, 3, 4, 5, 6, 7, 8, we were there as nurses.

I: Eight? Did you work in shifts?

R: We had the morning shift, afternoon shift and the night shift. We were changing. If you work the morning shift, the next time you work in the afternoon. The next time you work in the night. So, you change shifts.

I: So you were with two [others] in one shift?

R: Yes, two in one shift

I: OK, I understand it. And were there enough places for all the children?

R: If there were ..? Yes, if they took us from the ETC we had a passage where there was the street, and the ETC was here. And we of the kindergarten were here, we were different from them. We would only cross when the children were hungry. To tell them they needed food. We only come [to the ETC] to call them to bring food. We were not together. We were separate, where the children had lots of toys and ball and games. They were getting a [toy] motor car, [those] kinds of things. Those toys they were having and playing [with]. So that they would not be sad.

I: They were having more toys than they [would] normally [have] had…?

R: Yeah, they were having a lot of toys. The one they want to play [with], they take it and play with it. And when one [of them] got sick, and they have played with [a toy], we burn it, and give them a new one. So, we were clean with them.

I: So, they had almost the time of their life? And was it manageable since they didn’t see their parents?

R: Yes. Well, some of them they were [at] the age of thinking. They received it and called us ‘aunty, aunty, aunty, come’. When we got there, we came and asked them, “what is the problem, what is wrong?”. They said, “I want to see my mum”, “I want to see my dad”, “I want to see my brother”. So, we were confusing them. We were encouraging them. We said that their mum [had gone] to town to buy something for [them]. We said that “soon they are coming back. Go with your friends and go and play. Your mum is coming soon”. So, we encouraged them. When they come with their food we said this is from your mum. She said that you are hungry, come and eat. They started playing, so they forgot.

I: Yeah. But that was possible, that approach? They accepted it?

R: Pardon. Yeah, they accepted. When we said “go to play and go to eat”, they did it.

I: What was the maximum age of the children?

R: The [age range] was from new born until 15 or 18 years.

I: So, some children were already quite old? They understood everything?

R: Yes, most of them understand, so that a word of courage [was] needed. Then we made them happy.

I: So, what did you do when their parents passed away? Did you tell them?

R: Well we could not tell them at that moment. Therefore, they were discharged, we took them from the ETC, washed them, and gave them a lot of toys, gave them a lot of toys. We brought them to their residence. When they reached their home, we now told them, “your mother has died”.

I: The relatives told them?

R: Yes, [that] your relative has died. Your father has died. Then they were sad and started to cry. We would encourage them and left them in the community. [That] made them stop crying. Talk to the people and come back and give [the children?] to them.

I: One second, I have one question. In the community, they were not able to quarantine them?

R: They were able, but the elder people were [preferred?] the government was taking care of [them]. Because if they quarantine them, they were crying. They were confused and don’t understand. That is why we took them from the people to encourage them.

I: Do you know who came up with this approach. Who invented this?

R: Well, I don’t know that. I don’t know who invented that, [if] it was from the Red Crossers. But I don’t know. It is from them… the ministry of the government, I don’t know.

I: I am thinking if I know everything. I think I understand it. How is the collaboration with the ETC and the kindergarten?

R: Yes, they came to check us, they came to snap us. We got our own food. In the morning. In the afternoon. [At] night they give us our own dinner. They were always checking us.

I: And the social workers, they came there as well?

R: Yes, they came as well. They came to observe us, interviewing the children, the kindergarten [ones] that are able to talk. They were asking what we are doing for them. They were interviewing [us about] what we are doing for them. How [well are] we taking care of them, if we were good [at] taking care of them. Because otherwise if we don’t, they will be sad. But we did, they came and were taking snaps. And they went back.

I: Do you think you needed the social workers?

R: Yes, yes. For them to know those kindergarten people who were suffering, who lost their people. Unless we give them [encouragement]. That is why we were a made of them. To come and see. So they were the [messengers] to any ministry and the ETC [about what] we [were] doing.

I: Did you get a training?

R: Before I was working with those children we got a training at Pastoral Centre. Those whites. We came here. [After] 2 or 3 days we were here.

I; What kind of training did you get?

R: How to wear PPE, how to protect yourself. How to protect the children. How even to take care of the sick people [with Ebola?]. That even now god will [say] die. But now we are good taking care of you, so you will not die. You will get better, through the way of taking care.

I: Did you [also get] a training, because you are survivors, about how to accept your past? How to deal with your trauma from the Ebola?

R: Yes, they gave us a training how to manage our stress, how to deal with our stress. Embrace that to get the faith. We [have] got to accept that, it has become our way. Because it is through God. Because we had to interact with those community people, for them not to stigmatize them. To encourage them and to tell that this [is a] sickness. And that survivors cannot [infect] any other person again.

I: Did you get frequently refresher trainings?

R: with different organizations we got those trainings.

I: Which organizations?

R: Yeah, with the [government?] or with… yeah.

I: But was that because you were working in the ETC, or because you are a survivor?

R: Because I worked in the ETC? They did it mostly for the survivors. Not only [for me]. Not [because] you work in the ETC or not. You are a nurse you are not, you are educated, you are not educated. They call us. How to overcome our stress, how to control our stress. How to overcome our problems. Because a lot of the survivors as ladies, we lost our husbands. So many lost their husbands. They lost their children. They used to call us in these meetings and give us a word of courage. For us to overcome it. It is easier. Maybe God will give me, maybe God will have another husband and children for me. They always courage me.

I: But in the ETC you got never a refresher training?

R: We had [it] two times. We had two times another training here in Pastoral Centre. So, we came here [and] they gave a training to us.

I: What did they tell you?

R: They told us that this sickness [will be] overcome. The white people – that they are making chemicals. So that is like, is from God, the sick is from God. So, the ones that survived they know. The ones that did not survive, they are a kind of history. But as for me, I know that this sickness is from God. And I know that this sickness is a real sickness. Because some people, they are staying in their room. No person come there. No nurse come there. Nobody comes there. So, they don’t get infected and all of them died. Without touching them. So, they encourage us to share the message to other people how to take care of yourself. How to take care of the community and how to clean the community. How to take care and how to overcome your stress, that [is the] training they gave us.

I: And did they ask sometimes [for] information from you as a survivor? Did they involve you as a survivor in decisions?

R: Yes, when there were those people, the ones that survived, when they go back, they call us. Even before discharging they call us, because they are so confused, they don’t know what to do. They are crying crying, crying. They call us. We wear our PPE. We ask them why are you crying? Please stop crying, you have survived the Ebola disease. I am also here standing as an Ebola survivor. I was like you, lying here. But through praying, God has made me to succeed. So, if you have the faith in God, God will make you succeed. And you will stand as I am here today. So, we are giving them that courage. Some will think yes, if you survive I will also survive. And when they survive we will take them to their home. They call us. We come back and give a word of courage to them. Praise them. They will go with happiness that they have survived.

I: And sometimes you went to the community?

R: yes, we went to the communities with them. Sometimes we went with them and took them [back] to the community. We will explain to the community people that this person is a survivor. Thank God that he or she has survived. Thank God that your brother/sister has survived. Encourage this [person] as [any] other one. She is a survivor and will take care of others. Like all others. Please don’t discourage. If you do, you stigmatize him or her. So, you should stop. So, give courage to the communities of the survivors.

I: Did you always go with them?

R: Not always.

I: In some cases? In which cases?

R: Some cases. Like if this person lost everybody in his family, then we went with that person. So [that gave] courage [to] that community. But if it is a little boy or girl where the mother is alive, we carry him to the relative. But if no relative, we go with them to the community, for them to sensitize them.

I: Do you think that was the right way?

R: Yes, that was the right way, because if we went there, they know that it is right. Because some of the communities will think that we are coming from the ETC you have Ebola. If you come back, they still think that you have Ebola. So, if you come back they will be afraid of you, they will not talk to you, touch you, come close. They will be afraid. But if you go there and you will give courage [to] them, you sensitize them, telling them that this man or women will never give you Ebola or transfer Ebola. Because he is out of Ebola. You give them encouraging words. Explain to them the problem, and they will accept and will not stigmatize them.

I: But do you think you should have done it with every person that was discharged and went back to the family? Because I think it can be really important work. Do you think it would be better to do it with everybody?

R: No, why we haven’t done it with the children of the kindergarten, the government takes the social workers. The social workers were in the town. The government [sent them] to us. They went house to house. The government said if anybody stigmatizes survivors, they will give you a fine. You have to pay a fine [Leone] 500.000. That is the [order] of the government. So, we went to the communities to sensitize them. They said they were afraid. They said that they don’t have money to pay the fine. So, they won’t stigmatize you, they will encourage you. Because of the government. The government said that no one should stigmatize. If they do, because your mother or father has died, and you don’t have relative with you and they take advantage of you, the government gave that priority, [that] if they take you to the law, the law will take you. So, they will stop. That is why we took little children to their community, we don’t go there because the social mobilization people were there. It is only for the ones that [are] at the normal stage that have the chance to record something. We follow him to encourage and spread the message to them. The message that the government and their law will take you. Later the community social mobilization team will go there and [people] will still remember [what] we have said.

I: OK, thank you. Then I don’t have questions any more. Thank you.

**S4b Interview transcript Head dresser**

Interviewer (I): Pleun Welmers

Interviewee (R): support staff

I: Ok, tell me M. how did your experience start with the ETC?

R: Thank you very much. In the first place I have been a Red Crosser since 1999.

I: 18 years ago?

R: Yes, having done volunteering work in the Red Cross society I was fortunate to be youth officer for the society. Of course, voluntarily, Red Cross is a voluntary organization. During the Ebola epidemic, when we heard the information from Kailahun, I had to report the information to the branch manager.

I: What did you have to report?

R: When we heard the information I had to report to the branch head officer.

I: How did you hear it?

R: We had the information from Kailahun.

I: From whom?

R: We have my colleague, the youth officer, in Kailahun.

I: Ok, so you had contact with the youth officer. And then you had to tell it to the branch head officer?

R: Branch head officer. And he told it to the manager. That is the hierarchy, that is how the information flows. Around then, we found[ed] an organization - a psychosocial team. To go and talk to the community about how to control and to stop the infection. We [formed?] four teams, psychosocial team, social workers, etc. Right? But still there was an inflow of patients in Kenema Government Hospital. The Red Cross [can] only take up [an issue] when it is out of hands of the government. Because the Red Cross is extra to the government. So, the Red Cross came in and found the information from the national society. The national society [forwarded] the information to Geneva, Switzerland. There [are] the head officers. They sent ERU, Ebola response Units. Together with the WHO. They came [to] do the findings. After a week they said ‘ho, this is a problem’. We need urgent attention. That is the ERU. So, they called Geneva. Off course they came with all their gadgets. They sent a trip [delegation] to come and get [to] isolated place - what we call a camp [fakai?]. They came here with the French, the Spanish, a lot of different nationalities. Then when they were here [they thought?] the national society, they cannot do [it] all by themselves, [so] they had representatives. They had somebody who assisted in the management here. We had somebody that [specialised in] psychosocial activities, we had somebody [in] water management. And I was here and worked at the construction [site]. I always came here [to the] pastoral centre at six o’clock and we then [went] to the construction. In the camp. Right, during the construction. The lady who was here – her name [was] V.

I: She was an architect, not a nurse?

R: No, she was only an architect. She designed all the camps.

I: All the camps all over the country?

R: Yes.

I: And she was not from the Red Cross, because she designed as well all the other camps?

R: She designed it here?

I: OK, but in Kailahun, she didn’t design it.

R: No. From here, [she] only [went] to Kono, were we had another camp. In the process she told me earlier that after the construction they are going. But I wanted to continue. I needed advice from her. Where should I work? To reduce my stress?

I: Because it was really stressful? What made it so stressful?

R: Yes. Well the working condition here. Because having [to] leave home at 5:30 [am] to be here at 6 o’clock and being back here at 6 o’clock [18.00?] and go home. It was a serious job. They needed assistance. Without us the white people will not satisfy [their obligation?].

I: So were you the only one who helped in the construction?

R: Well in the construction I was taking people to help us during the construction. But I was in charge. So I go, we want [a] hundred people here to do the brushing - the community people. I go to the chiefs. I collected the community people. That was why we know those communities. When I need casual workers or volunteers I go to the chiefs because that was the argument laying down. We should give them priority. In terms of jobs. Because they realize ...

I: They helped you and you should do a favour back?

R: Yeah, that was so. After which I reached her and she advised me to work in the dressing room as a dresser. Because we constructed all the locations. This should be your office, you have all the materials here. You will not even [be] in PPE, you [will] only instruct other people to dress and go to the high-risk and go to the undressing room. If you want you can go to the undressing room. If you don’t want to, you assign somebody. You will be the boss. I said “that is good”.

I: To have a little bit less stress?

R: Yes, that was why I managed the work with the ETC. In the camp there I was also supporting the office here. Because all the workers were also youths.

I: They were quite young?

R: Yes

I: What is young? What do you consider “youth”?

R: They are youth when they are below 35 years.

I: And above?

R: 25 years

I: OK.

R: The majority is below 35. Of course I am above 35 years.

I: Luckily, I am a youth. I am not below 25, because what do you call people below 25? Toddlers?

R: So right! That is why i managed to work as... By then I was asking a lot of questions. As dresser who is dressing who? Is that my job? They said no no no, you are never seeing the patients. You are only dressing the staff that are seeing the patients. That is my role. She explained to me they could call us for a training. So, during the training where do you ask questions? For sure I was above everybody, so I answered automatically the expatriate that came here. “This man should be the supervisor of the dressers”. So, I became the supervisor. Then I came here. There were four teams. I was with the management now.

I: Sorry, can we first go back to the training?

R: Yeah

I: OK, what kind of training did you have?

R: Yeah, in the training.

I: Who gave you the training

R: The expatriates.

I: And what was their job?

R: We had doctors, we had nurses, we had IPC expatriates. Yeah. We had people from the WHO, who gave us criteria.

I: All these people, doctor, nurses, IPC’s - they gave all the training?

R: Yeah. Because they said this is an epidemic we need to know a lot before we go to do the job. Before knowing we need to protect ourselves before you protect somebody. The priority there was to protect ourselves. So we had a two weeks training there.

I: Two weeks? Together with all the other IPC? What did you learn within this training?

R: We learned about hygienists, how to [run] the ETC

I: Can you remember it day by day? Or not?

R: Yeah.

I: Please tell me. First day

R: First day was the introduction.

I: Of what?

R: Introductory - the overview of Ebola. They gave us an overview about the epidemic. They even taught us the different types of Ebola. That was all in the introductory part. Then from there we went to the hygienist practices. Because according to them it was the paramount [aspect] of all the management centre [tasks].

I: That was the basic?

R: The basic. Then we went to the PPE.

I: This is still on the second day?

R: That one we started but we continued on the next day. Because the use of PPE, the people had to demonstrate. I was there to direct and to give the instruction. To see when it is correct and when it is not correct. To see if there is no hole. They are going today to the high-risk. So they did it over again [for] the understanding of everybody. Right? Then we even go to the management of the patient. Because if you don’t use the PPE correctly you are not able to manage the patient.

I: What do you mean with to manage the patient?

R: When they come with the patient. How to encourage and how you take care of the patient. Like the dressing the patient got. Because the dressing that they were using when they enter. We had to change. We had to give [them] another dressing [set of clothes?]. That is why we had a lot of [clothes?] in the stock. Yeah. That is why at any time we had new patients they called for dressings. So, [that was] all that we were trained on. Even the feeding method. We were all trained. Together with the nurses. Because the hygienists they were, the IPC. The nurses cannot go without the IPC staff.

I: OK, so on the third day the nurses, they joined you?

R: Yeah. Of course the nurses did their training first. When we were doing the IPC training we joined the nurses also. When we [were] doing the management of the patients because we do that together. Of course, that area, the head nurse was responsible to do that, together with all the other nurses. Of course we were all interacting now with each other. Actually it was a nice organization because we did not work [in] parallel. We worked together. That is why we were security to each other. Whenever someone saw there was a mistake someone [will] pick it up. You said ‘that mask is not completely covered, get out’. That was what we were doing.

I: OK, and then the fourth day.

R: ha ha, you want to go through everything. Of course we were going back and forth,

I: Between pastoral centre and the ETC?

R: Yeah. Of course [on] the fourth day we took them to the ETC.

I: For the first time?

R: Yeah. To see how the centre was constructed.

I: Because the ETC was already finished?

R: Yeah, to see how the place was structured. Fortunately, and unfortunately for some people, on the fifth day we had the first patient. That was the day people decided to appoint some people to sleep there. And I was among them.

I: You slept at the ETC?

R: Yes. Because, I was dressing the staff to meet the patient. And without me [being] there [to] get the experience, they will do something wrong. That was why I decided to go there and slept there. The other day they started to bring people from the governmental hospital. The other day we really started. Half of the logistics [was] brought [to] the camp there. We started working there. It was very nice.

I: And then the next week. Because we are now on day 6. How did it continue? Did you still keep on working in the weekend?

R: Yeah, yeah. OK, of course. It was a health system.

I: I understand, but you have not completed the course or training, and there is only one patient. I can imagine that the expats [were] doing that.

R: We [were] doing it with them. The other week also. We decided to divide the shift now. So when people were doing the training here, there were other people that got training here. Since we had certain patients. The other day when people were doing the training, other people were going to their work. That was how we were doing.

I: OK, and do you remember on the sixth day what you learned.

R: That was the day we separated the teams.

I: Yeah I understand, but what did you learn?

R: That was the day we learned about the dead body. Of course, everybody knows [what] they will be doing. Because I cannot be a dresser [only?] since I am a dresser and I know I cannot correct her. That was why we trained everybody together. So the sixth day we trained how to manage the dead body. How to clean the death body, how to use the materials to the stretchers to the mortuary and the life. That was...

I: And the seventh day?

R: Of course from there we had on-and-off trainings.

I: Yes I understand

R: The third day of course, I have already explained what the dressers have been doing and the hygienists and the waste [managers]. Of course we had a waste management team.

I: Waste management team?

R: Yeah, they take care of all the waste.

I: But who is it?

R: The hygienists. [From among] the hygienists we decided to select somebody to take care of the waste, even [waste] food, [we had] people responsible to do that. In the kitchen [they are] the people who go there. Before the cooks go there. And when the hygienists go there before the cooks or the supervisor will go because they put it in the big dish. Until the supervisors go. Because we cannot leave the job and all together take the lunch. When we go in there, some people take their lunch. Of course, we had a big hole where we used to take our lunch. We had a big hole and we go there and some time when we follow. Yeah. Then [there is] the entry to the ETC. We had steps to follow. When we go from town, of course we come all together. There is a park where the vehicle stopped, everybody [gets down] and the first entrance is the place where you leave all your dressings [clothes] from the town. You meet all the scrubs that we are using. You leave all the stuff that [has] come from the town. Use the scrubs. And from there we have the sprayer. After you dress, the sprayer sprays your boots before you enter the low-risk. Before your come out from the low-risk, they spray you, you take your home dressing and you go out. The scrubs - you cannot take [these] outside.

I: OK, but you take your scrub out. It is considered a bit risky. But was it not a risk that the infection went from your uniform to your own clothes? Or did you run naked to the next room?

R: The scrubs that we were using were only used in the low-risk.

I: OK, so it was not risky. You could still change yourself in the same room as you left your uniform?

R: Yeah, as you remove the scrubs you are not using it any longer. The hygienists will take it to the laundry.

I: OK, I still don’t know the 7^th^, 8^th^, 9^th^ and 10^th^ day of the training.

R: The actual training we did it for four days. Then we were…

I: Yeah, and then the patient arrived, and on and off you had the last part of the training [on the] 7^th^ day; what did you learn?

R: We had to refresh.

I: And the 8^th^?

R: Also refresh.

I: And the 9^th^ only refreshing. The whole week refreshing? Only refreshing?

R: Only refreshing training. Doing the practical exercise. We had a few exercises. The use of PPE, the use of other materials. We had to demonstrate all the things we had in the ETC. That did we train.

I: OK, then you started working there. I understand how you did with your clothes. Did you have refresher trainings often?

R: Oh yes. Of course, in the dressing room I did often refreshing training with the staff. Sometimes in this trauma healings sometimes people come from different communities and homes. Each time people come here in the dressing room I train them. I sensitise them. I counsel them. We always do counselling. Because the use of PPE it is very hot we need to counsel them. You cannot use PPE more then 45 minutes. I told them all this. You cannot use PPE as [if they were] normal dressings. When they enter I noted the time there they were going [in]. In my book. I had a book in my office. That one, I introduced that one. That is why I asked the expatriates to have a clock. I had a clock in the office. So when it is time, I call the supervisor. Hi, supervisor, the last one that went should come out now.

I: And that went [on] often?

R: Yeah!

I: Yeah, that happened very often? That they didn’t come out in time?

R: Often. Unless I was checking.

I: They didn’t come out by themselves. You had to call them. So you had to check the time? All the time.

R: Yeah. Yeah. All the time.

I: Did you sometimes forget?

R: That is something you cannot forget. That is why I told the other dressers.

I: That is why you were not alone doing that? Others did do it as well?

R: Yeah.

I: and you checked the other people. And did other people check you?

R: Oh, yes

I: Who?

R: The supervisor, the other supervisor checked me. Even the expatriate. When we are together. At one time, A. decided to go and check whether I [am doing] the correct way of dressing. She went to most of them. She said “Musa, I want to go in, what do I need to do?”. You want to go in? Stay there until I am ready for you. She stopped. “And now go and wash your hands”. I commanded her. With all the procedures I dressed her. She was following all my instructions, because I was instructing her. Take all this, take using. And then she said that she didn’t want to go in. She wanted to undress. I said that this was not the place. Go to the undressing room. There you are going to undress.

I: OK, and the refresher trainings, how did they go?

R: Well, when we were in the ETC, of course we called the expats to do the refresher trainings. We do the refreshment trainings. And in the refresher trainings of course I was doing the lectures on dressing, because I was in charge of the dressers. I was doing the refreshing on and off. To [ensure] nobody [will] forget what we have done. Because sometimes some people do forget. That is why we do the refreshers. Of course, they were changing shifts.

I: Ok, you gave the training. Did they ever change the procedures?

R: Change the procedures? Well, when there was a changing of procedures from the expatriates I was there. I call all the other workers, we were in different teams. I called the other dressers I tell them [about] the changes, train them on the method they have changed. And go to their different teams.

I: Did they change [procedures] often?

R: Well, It depends on the arrival of the expat. Sometimes when the expat comes, they were observing how we manage ourselves. But the criteria…if there is any change, he asked us, why are we doing it. We tell him or her the reason. They do not change [arbitrarily], they ask us. [More, it will be] “suppose we add this that?”, or whether we know this one is correct or this one is correct. It [is] sort of saying not that we are doing wrong. We have this one and this one, can you see and compare?

I: So you understand the improvement?

R: Yeah

I: Do you know why they didn’t do it from the beginning? Do you think the new expats had another training?

R: Well, initially we heard from them that Ebola had no treatment. We only manage the disease, there is no treatment. In managing the disease we had a lot of methods. So when somebody comes with his or her own idea, we take it because we are managing. Right. So that is how we [were] organized. And everyone that came with his own idea, we checked, and if it worked better, right we did it. Until others come with their own idea and we see it. If we see it that person comes is different is boring to us. You are quite correct, but this one is it not cause-effective [?]. That is it. Putting ideas together.

I: So, you were well involved in the decision?

R: Exactly.

I: And was that the whole dressing team, or was it only you?

R: Well, that is why when the supervisors had a meeting, they called me. I was in the supervisor meeting. And most of the decisions were taken in this supervisors’ meeting.

I: They were making the decisions. Who was in this supervisor meeting?

R: Exactly. Like the local staff, the local supervisors.

I: Like you, and M.?

R: Exactly.

I: Besides the supervisors, [was there] someone else?

R: The team supervisors all together take decisions.

I: But no expats were there?

R: Well, when the expats come, if it is general, they call the head nurse and the head supervisor. They always [had the] head here. When…

I: The expat head nurse?

R: No.

I: local, J.?

R: And G.

I: who is G.?

R: The head of the IPC. They are always in between and they are always working together. The head IPC and the nurses. Right. When they have information from the expats here. They go and call the supervisors. The team supervisors. Including me. We go and meet at a certain quarter. We share the information and go to the teams. I go with the information to the dressers. Others go to the IPC’s. Everybody, different, different quarters. Now the information is spread.

I: And when you took the decision, you talked with the expats about that?

R: Yeah.

I: So you took the decisions?

R: We took the decisions. Because the expats saw that we were doing a very good job. We were managing the ETC. They also relied on us. We took the decisions. That is why we decided to change M. (head dbm [?]) and bring [in] G. Yes, I can remember now his name.

I: But the expats came with the problems. When there was a new expat and he saw an improvement, that was not made in this supervising meeting.

R: No

I: What kind of problems are spoken [about] in these supervisors’ meetings? Is there a notebook [minute book] of these meetings? Do you know that?

R: There was a notebook

I: Do you know where this notebook is?

R: I will check it. I think I had some notes. Most of the training we have been doing we have it in our [data] sticks.

I: You have put it all in your computer? So All the notes are on a computer, or on a stick. Can you maybe check it?

R: Yeah. Yeah. Yeah

I: That would be perfect. Because I am super curious. We will go in depth about it when you have checked if you have these minutes. Do you have any incidents you can tell [me] about? Where there many incidences?

R: Yeah. Yeah there were some. At one time there was a patient that wanted to escape.

I: Did you make a [notification] of these incidents? Because I know in The Netherlands we have to make reports, and if the incident was super bad, we had to inform the inspection institute about the incident.

R: That is why we have notification. We can do it.

I: You made a notification on paper?

R: Well we wanted [to]. That is even why I wanted A. (social worker) to come [to this interview] because he has everything.

I: You think he has these notifications? Of the incidents?

R: Yeah. Yeah. I spoke with him the last time actually.

I: Do you think there are incidents that are not reported in here?

R: The ... incidents … that are not reported …?

I: Ok let me ask you another question [ask in another way?]. What kind of incidents were frequently happening?

R: Well, a certain time ago, of course I was always at the ETC, even if I had a day off. Because they sometimes called me to be around [in case] there was any incident. **We had a mad [person] who forcefully wanted to enter the ETC. He went to the security “I have Ebola, I have Ebola”. I said “no you cannot come”.**

I: That is what you said? You were at that time at the gate with the security?

R: Yes, where my office was, was very close to the gate. I overheard the discussions. The security called me, “Mr M., come, somebody is coming to instruct us”. He said **“I have Ebola. I want to go and lay down. He said you don’t want supply me, that is why you don’t accept me”.** It was very amazing. Ha ha ha.

I: Ok, some people take it from…

R: But what happened? I talked to him.

I: Because you could see he did not have Ebola?

R: Fortunately I know him. Even the nearby village where he comes from, they know that.

I: **Ah, he just has a mental issue?**

**R: Yeah. Mental problem.**

I: You knew already about him?

R: Yeah, I knew already about him. You have to counsel him and give psychosocial support. By giving him something to eat. And when he had something to eat, he left the plate there and said, thank you very much. Ha ha ha.

I: Thank you very much, M.

**S4c Interview transcript Cook**

Interviewer (I): Pleun Welmers

Interviewee (R): support staff

I: Can you tell me how you started with working for the ETC?

R: Application.

I: Did you see an ad?

R: No.

I: How did you know about it?

R: Well did the application because I heard the announcement. They had come to take a job at the ETC. I passed [the interview?]. I am the head cook of the ETC.

I: I heard about that. How did you hear the announcement?

R: From my friends. Then I told the Catholic bishop. He is my brother. They told me yes. I called from Pastoral Centre [to ask] if it was true.

I: You were not afraid?

R: First day I was afraid. I was afraid. But as soon as I saw my companion, then I felt free.

I: So, you passed the application. Did you do a training.? Can you tell me a bit? What did they teach you?

R: Yeah. Yes. We came here for the training for one day. How to prepare food for the sick people and the workers.

I: They thought you didn’t know that? Because you are cooking already at home.

R: Hah hah. They interviewed us, and they told us how to prepare for the sick people. So, I asked what kind of food [they] would want me to prepare for [them].

I: Who did you ask?

R: The sick people. The patients. I asked the patients. They told me the type of food and I prepared that.

I: OK. At home you cook every day, right. What did you learn, except from what you knew all ready in this one-day training?

R: One-day training. They taught us about Ebola. Then when we went to the ETC to work, we had [a] place, we had to stay there, we could not go to another place. Only when you go home.

I: Ok, I understand. Who did you [do] this training with? And then, after the training how many days later did you start to work at the ETC?

R: Everybody. [?] After the training we started to work after two weeks. Because we had to prepare for the kitchen. Prepare everything what we need in the kitchen. After that, after two weeks, we started to work in the ETC. During one week.

I: So, during one week, you prepared the kitchen, one day after. And there were two weeks that you didn’t do everything [anything?].

R: Yes, because we were setting the fire and what we needed in the kitchen.

I: Because there was nothing, and you had to organize it yourself?

R: Yes. Yes.

I: And at the first day you were already a head cook?

R: We cooked the first day. When we ate this thing.

I: You were the head cook already? They asked you in the training to do that?

R: Yes. Yes.

I: Can you tell me about the activities during the day. You started in the morning?

R: In the afternoon. The way I cooked for them?

I: [How?] did you start with on [any?] random day.

[translating my question in Krio]

R: They gave me money every week. During the week, they give me money for the week. Thursday to Thursday. I used that money every day for the afternoon and morning shift and also for the patients. I prepared the dinner for the patients. I prepared tea and bread. In the afternoon around one, I prepared rice and soup for the sick people. For the workers we prepared rice and soup. Only for the patients we prepared three times a day.

I: in the morning you gave tea and bread. Did you make the bread yourself?

R: No, I bought it in the market. They gave me money to buy it. Because they gave me money for the week. I bought every day. From Thursday to Thursday.

I: So, did you go in the morning to the market? What time did you start?

R: Yeah. 8 o’clock. I went to the market, to buy everything. Then before twelve I will be in the ETC. To prepare the afternoon shift, for the afternoon people.

I: So wait, the bread, at what time did you give that? But around what time did you give it to the patient?

R: The bread. I bought it in the morning. I gave it at 7:30

I: Ok, so you went at 8 to the market and at 7:30 the patients got their breakfast?!

R: If I bought it like yesterday, I bought it for the next day. I left it in the ETC. Then the people stayed in the ETC to prepare the breakfast and I went to the market to buy the bread for the next day.

I: So, they got bread and tea? And the bread, how did you serve it?

R: The bread, we put sardine inside the bread, or butter or mayonnaise. Not empty bread.

I: And then, did they get a whole bread.

R: One for each patient.

I: How did the patients receive it?

R: [It was] not me giving the bread. I gave it to the nurses. They gave it to the patients. I only prepared the tea and bread.

I: Did you put something around it?

R: We covered it with ancava cheat [?] (a kind of plastic). And the tea we put it in the rubber [flask?].

I: How many times did you cover each bread?

R All the bread. One was enough.

I: You gave it to the nurses. Did you put on a desk and they took it?

R: I put it on a table and the nurses, they took it from me. I put it one by one in a container. Only the tea in the tray in the cup.

I: You put it already in the cup?

R: Yeah, we mixed it and we put it already in the rubber cup.

I: It was not in a Thermos jar. They didn’t have to pour it anymore? But was it not cold when you arrive at the patients?

R: In a cup. The distance was not far. So, it was still warm

I: OK and when you gave the breakfast, you directly started preparing the lunch?

R: Before we gave the breakfast, we [had already] start[ed] preparing the lunch. Then in the afternoon I bought mangoes and we gave fruits. For the patients, only for them.

I: So, the breakfast was only patients, [but] lunch for everybody? For the lunch, you prepared always rice?

R: Yes. We prepared rice, cassava, fufu; we changed it. We cooked pap, you know it? We cooked porridge for them.

I: Was [it] everyday scheduled what you [were] to prepare or did you go to the market [to] see what was cheap? How did you decide what you were going to make?

R: Before I went to the market, I had a list. I prepared everyday what I have to cook; cassava leaf, potato leaf, etc. I wrote it down. And then I got money

I: So, they gave everyday money?

R: Yes, nooo. They gave me money by week. Thursday to Thursday. If they gave me money on Thursday, the next Thursday I got new money.

I: Ah, OK. So, you thought what you wanted to make, you made a note of that, you measured how much it will you cost, and then they gave you the money?

R: Yeah. Yeah. Yeah. Yeah. For the provisions, different money. For what I need to prepare, different money. For the wood, I asked for money. I asked them, and they sent me money for the kitchen.

I: How did you decide your week’s menu?

R: I decided ….

(Translation.)

R: I got first the money for the week. And it was enough. I made all types of food. But the money they gave it on Thursday. Inside that money I have to prepare different types of food. It was enough for the week.

I: [How did you make] this schedule? Why did you decide on Monday cassava, for example, and on Tuesday potato leaf?

R: Because we change the diet. We changed, so not every day cassava leaf. When I prepared cassava leaf, I have to change it. Not every day cassava leaf. We have to change it. Not every day one sauce.

I: But this was just what you wanted to prepare?

R: Yeah, the workers liked them. Anything that we prepared for them, they wanted.

I: Did you ask the workers; what do you want.

R: No, only the patients. Because they are sick people. So, you asked them, what would you like to eat? During the morning. If it was cassava or fufu. But the workers wanted every type of food. Because our food was delicious. They liked it.

I: So, you asked the patients on Wednesday, what they would like to eat for the next week? But there is a possibility that they are not there anymore the next Wednesday.

R: I asked them during the evening, every day before finishing work.

I: But if you asked them, it is possible that it didn’t fit within your budget anymore? Is it [the case]?

R: No, because I almost [?] had the budget. I asked them every day, Thursday to Thursday. I asked them throughout the week. When they discharge patients, other patients come, it [the ETC] will not be empty [of] patients. The new ones, when they come, they get the same food. When the people are discharged, they take the food. And they don’t complain.

I: It worked in that way?

R: Yeah.

I: And how did you give the lunch? Did you give the pan to the nurses, or in a package?

R: We put it in a package. And then we put it in a big bowl. Give to the nurse. They carried them and supplied the food to them.

I: And this tray, did you clean it?

R: We cleaned it every day with soap and [sapo?].

I: OK. And did others clean it with chlorine?

R: Yeah, the water that we used was with chlorine. It was already mixed. Every day, the tap water.

I: Did you have different kind of coloured taps?

R: Yeah, we were using the three different type of taps.

I: So, did every [one] used the orange and red one, or only the green?

R: We used red.

I: For what?

R: When washing the pans. The things, we washed it with red.

I: Why with red and not green?

R: We only used red because it was not too powerful. The chlorine was not too much.

I: Did you use the fresh one later?

R: No, we didn’t use another fresh water.

I: And in the afternoon, you prepared the dinner? What did you make for them?

R: Yes. For the workers, or [do you mean for] the patients?

I: For both, they get the same [entitlement]?

R: Yeah

I: What did you prepare?

R: Different sauce

I: So different sauce, but the same rice? So, you had for the whole week 14 different sauces?

R: Yeah.

I: For every day different sauces?

R: Two different sauces.

I: And the division worked the same as [for] the lunch?

R: Yeah.

I: Oh, and you were talking about the fruits. They got fruits? Only the patients, or the workers as well?

R: Yeah. Yes. Only the patients. I bought fruits for them. Different type of fruits.

I: Everyday different?

R: Every day, and the nurses gave them.

I: Did you already cut it [up]?

R: After washing them, put it in the bin. And the nurses came and gave it one by one.

I: So, it was not packed?

R: No, but we had to peel it. If it is orange. In the kitchen. We put it in the bowl and give it to the nurses.

I: So, this bowl, did you wash it with the red tap?

R: Yeah, we washed it.

I: OK. And in the evening, what time did you finish?

R: 7:30. We waited for the bus to carry us.

I: What time did the bus leave?

R: 7:30, in the evening.

I: So, you worked like from 8 in the morning till 7:30 in the evening? And did you have any free day? And the others?

R: Yes, yes. For me, for the boss, no. The others had free time. One free day.

I: Each, one day. Even when it was not a busy time?

R: For me, I have to work every day. Even on Sunday I had to work every day.

I: And where was the storage of the food? When you had [bought] the food, you brought it to a store?

R: The stor[ing] of the food [?]. When I had bought it, I brought it in inside the ETC.

I: That was in the low-risk area?

R: Yeah, in the low risk area.

I: And did you get a training since you went in the low-risk area? Did you get a training?

R: I entered in the low-risk. I directed the store. After taking my food [to the] store, [and] then to the gate, they spray my feet with chlorine. And then I went back to the kitchen

I: So, what did you wear in the low-risk?

R: I had to wear my own clothes. And when I entered I had to use the boots.

I: So, you were in your own clothes and you changed only your shoes?

R: Yes. Only the staff, the workers, but for [us] in the kitchen [we] wore our own clothes.

I: OK, and then [when] you went to the store, how could you carry everything?

R: I put it in a big bowl, everything we need. And someone helped me to carry it to bring it into the kitchen

I: OK, and did it have wheels?

R: I put it on my head.

I: OK, and one time going back and forth was enough?

R: Yes.

I: And you changed only your shoes. Did you have own boots?

R: Only shoes. No, I don’t have boots, I used the boots of the ETC.

I: OK, and then you went out and you brought all the staff to the kitchen.

R: No, from the kitchen inside they sprayed my feet, my slippers, then they took the boots, [and I] wear it until [I reach] the store. From the store, [I] come back, [they] spray my shoes, and then to the kitchen.

I: OK, and the plates, did you get something back [from the ward] that you needed to clean?

R: Only the workers, after [they] finish eating, they put the plates in the big rubber. And [we] come and take it, collect it.

I: So, you went in the low-risk again to come and collect it?

R: Yes. The canteen was outside the low-risk area. We had a big rubber in the canteen. After eating they put their plates in the rubber. Then we came and took the rubber, and entered the kitchen to the tap to wash it.

I: Which tap did you use?

R: The plain one, the red.

I: OK. So, if you use the red, did you wear cloves to protect your hands? So, you cleaned it with water and chlorine?

R: Yes. And soap.

I: And soap. And then you used fresh water later to …

R: … to arrange it.

I: OK. But you never got a training about how to perform in the low-risk area and what the risks were?

R: No, no training. Never.

I: Were there ever any incidents?

R: Incidents?

I: [For example], there was a mistake, [something] happened, or you had to improve?

R: Yeah, **someone died** [follow up - natural causes?]

I: In the kitchen?

R: No mistakes.

I: Not at all?

R: No.

I: And did you ever change procedures? That you had to do things a different way?

R: Before [at first] we wore scrubs, but then they told us not to wear them, because we were in the non-risk area.

I: And did they change anything else?

R: No

I: Can you tell me something about how you prepared the food for the workers?

R: We made the food and we put it on the plates. Then [we] put [it] in the big rubber and they came and received it. From the table, anyone [can come], and take a spoon. After finishing [we] come and replace.

I: So, the rubber?

R: For the workers, no rubber. We have to put it on the table. It is a very long table in the kitchen.

I: So, you put the food already on the plates?

R: Yes, and spoon.

I: So, they didn’t have to divide it by themselves, you did it already,

R: Yes, we did everything. Just put the plates and the spoon. They will come and take the plates. Every day in the morning and afternoon.

**S4d Interview transcript Head supervisor**

Interviewer: Pleun Welmers

Interviewee (R): medical staff

Replaced people [because]:

- Some people did not follow the hygiene [rules]
- Drinking
- Fighting

1. The burial supervisor was replaced. He did not follow the rules. And he was argumentative. He took a bit of alcohol and it was hot in the PPE so he was influenced.
2. Another person, she was a supervisor and she took a swab but she was not allowed to do.
3. One was stealing from the high-risk area. The boots.
4. One colleague was in ETC. She took this phone with her in the high risk [area] when she stayed there. Before the happy shower [?] she gave someone her phone to take it out of the high risk [area] so she could keep it and [that] was not [allowed]
5. Someone that used a wrong name. Many people came and did their job interview, but never came back anymore. They were so afraid. There was a man that saw a name of someone that had done his interview and introduced him with that name. When he had to hand in all his official documents, they would have seen his wrong name. He went to Freetown to change his name. But colleagues were informed about his travel and told the story to the federation. He was fired.
6. He took the knife from the IPC
7. Had direct contact with a patient. Everybody saw he didn’t have right PPE. He had to be in quarantine but he refused.

This man knows that 4/5 people were asked to be in another unit, since they had some problems with some jobs.

The Head Supervisor [said] that if expats did not look, rules were not always followed. For example, that you had to clean your plate with 50% [0.5%?] chlorine and then give it back to the kitchen. When expats were around, all rules were followed strictly. The head supervisor managed as well problems that appeared. If people did not work well, sometimes he did not inform the expats, but talked to the workers himself. So there was like a hidden atmosphere. The expats did do exactly the same. For example, once a white nurse was sick and went home after a couple of days [after?] he arrived. He lied about that he was sick. In the end he had malaria, but the expats did not want to bring panic in the ETC. There were more such a situations. He does not know if the expats knew about their [Sierra Leonean staff] secrets.

Laundry service was not around [during] the first two weeks. They used disposable once. This was too expensive.

Supervisors were responsible if there was enough stuff at the stores. They never had shortages. They had only sometimes [did] not [have] the smaller sizes anymore. So, they used tape for the gloves. The warehouse officer had the responsibility to ensure there were enough.

Team supervisor had phones to communicate. Radio was available for the ambulances. But in the high-risk [area] [there were] no phone nor radio. You could talk to each other.

Absence of the head nurse. First she was not supposed to go since they could not miss her. But later she went. She came back earlier than planned since it was not running as well as meant. He says that there was two people in the hierarchy that were not around. So people did have less possibility to report and chat about situations. More often people were not around. They went more often ‘alone’ in the high-risk area.

Quarantine, 13 people, 7 nurses and 6 IPC’s. Some people refused to stay in quarantine, they were dismissed. All people were affected from 1 shift. There was a shortage, but you could not train new people. Soon fewer patients came, [because] Kono’s ETC has been opened.

Social workers were in charge of the visitors. They could not go to the high-risk.

Below PPE was your uniform. This was not a problem that you wore it as well in the high-risk. Only if you sweated a lot you had to change.

The supervisor managed the division of the jobs. At the beginning you had all your own division but later it was rotated. The supervisor looked after [things to ensure] that everybody got to do all jobs. But he coupled the good with the ‘new’ once. He has to talk very kindly about that. You want that they are still satisfied.

They got a call that someone passed away. The specialist medical officer (an expat) confirmed the death. The dead body management team went after three hours, and they discovered that the person was still alive. From that moment they took the body out of the tent and left it in a place close to the morgue. Just to be sure that the person is put in a body bag is not alive.

Another incident that happened with dead body management. They got the call that someone passed away in the confirmed tent. As soon as they arrived there, they discovered that it was the wrong tent. As long you have entered the confirmed tent, you cannot go back any more to the other tents. That is what they did; they went back to the other tent to pick up the body there.

IPC delicator [director?] and the head nurse evaluated directly and if possible, they improved the procedures.

A boy came out to the low risk. He was eight years old and was just hungry and bored. So he was walking around.

A patient tried to rape another patient. This patient was not sick. It was in the night. So the nurses had to take this patient.

Once one of the hygienists found a sharp object in the bin. People are lazy so one of the nurses did not throw it in the right box. The supervisor said it to the head nurse, so they fixed it without the expats.

There was a division between the volunteers and the workers. The workers were really driven by the money they earned. That was their motivation. Complained a lot about the money and when they get it. The volunteers were patient to get their money. They were already working before without money. Later they became the same as the volunteers [workers?].

When the workers were hungry the went to the kitchen and almost jumped in. They were asking for food and it was hard to satisfy since nothing was ready yet. Delicates saw it and they made a two-barrier fence between the kitchen and ETC.

Africans [find] it really difficult to follow a rule. They are very lazy. Because it does not have to do with education. We are faster. If you tell what to do, it will come out as a defect. They don’t see that it has a consequence. When the expats are around, then they can follow the rule. [No?] jewelry, plates, washing your hands before spraying, closing the gate,

Most of the times he [the supervisor] kept the things that he fixed secret.

Rounds to the patients were done, sometimes just to talk or give drinks.

20.00-08.00 is the night shift. Only when patients were asking for help, the nurse went inside. The rest of the time they were waiting. So, for the nurse there was some work to do, but for the IPC less.

In times of fewer patients you were working on stand-by. Morning is 08-12.00. At the beginning [it] was until two. And then 14.00-20.00. They changed it, to divide the work more equally. The morning [shift] had most of the work to do.

**Additional notes from the interview with the head IPC supervisor**. He was telling this story after the recording was stopped.

There was an issue that an expat was bitten by a patient. They kept it secret and the patient went back to his country. They kept it secret to avoid panic [among] the locals.

If it happened often, he didn’t know, but he remembered another incident. There was an expat nurse and he was cleaning his own vomit. He met many of the Ebola symptoms. He denied to the locals that he was sick. However, he was brought to Freetown for further research. This is what the driver told the head IPC supervisors. This expat just disappeared and locals were not informed. He was new in the team so not many people would have missed his presence. Later the head asked about this case to the expat and he told him that he had malaria and went back for that reason. They didn’t want to bring panic in the ETC.

**S4e Interview transcript Sprayer**

Interviewer (I): Pleun Welmers

Interviewee (R): medical staff

I; So maybe you can tell me a bit about you started to work at the ETC?

R: I have been a volunteer for the Red Cross society from 2008 until now. So in **2014, May the 18^th^** we were called through the office. We got the information before the Red Cross have put the advert. We receive it first before the media. I was having a job where I was talking to people going to houses. But then I was called by the Red Cross. Something like.. you can [see] this.

I: Ah, you brought it with you!

R: No, this is different, this is about malaria.

I: Oh,

R: This is how the card was. A hard card. That was having all the important information towards Ebola. Towards that time when we were having the community we started to talk to people. After which we were beginning to be interviewed about the building of the ETC as Sierra Leoneans. And give the intention to them. And after it later we received IFRC. Our brother was there with the centre there at the construction. And we gave another assignment then to move around the centre. Talk to people. They need to accept the way how it is, it is natural. And as it [has] come, we put things in place to see that things are irrigate that. Thank God they were following the steps we were telling them. Especially the people around the ETC. They follow most of the steps. After that we came we go to the ETC to start to work. We were called for orientation how was the plan and how it exists. Later [they] call up a training for us. We were trained here in pastoral centre. We were trained [in] varies activities. One was hygienist and sprayer, dresser, undresser, maintenance, the nurses were trained. All were trained here. And then when all of us were trained, we moved all of us to the centre. First the centre they start to separate the first badge [batch] and the second badge. And we were among the first badge. We went to the ETC. So that was how I was involved.

I: Ok, so first you went to see the construction and then you got the training. Can you tell something about the training, who did you get it from? How long did it take?

R: Yes, construction. Well the training was held by the facilitator called Mr. James. He was from the Sierra Leonean Red Cross society,

I: He was from the Sierra Leonean Red Cross society. Ok.

R: Yes. Then there was a person who trained us even though he was having another colleague that trained us. He trained us on the same exercise. He was the forefront person.

I: Do you know the job, his background within the RC?

R: Well from what I might understand. He cannot be a nurse, but he has some medical background.

I: He told you everything in the training?

R: Yes, he [did], but he is not a master at everything. He has his specialisation and other people that are coming up. So, it kind of a little [changeable?]. So, there were other people who were with them to see that everything is going correct.

I: So, he organized the foundation of the training and other people did the trainings from their specialisation.

R: Yeah. Some were expats and some not.

I: What did you learn in the training?

R: I learned the skill of spraying and all of the other things to be an IPC. Because this was the general training. At the beginning. Because you cannot go to the ETC with[out] having the knowledge how the ETC is operating. So, all the knowledge within the ETC was given to us with the exception how to give the drugs. That was not given to us. All of the words that they were saying to us, that was keeping the centre clean, that was our activities. We will learn how to do it. It was all our area of specialization. Like if I am dresser, you cannot go in as a dresser if you don’t know what the layout is of the ETC. You should know before you go in. And to who you have to do it.

I: How long did the training take?

R: We were trained for about a week or so.

I: Did you get this training with other people or only with the sprayers?

R: Yes, we were trained as one. Sprayers, undressers, laundry, dressers, hygienists, DBM. We were all trained together.

I: And after one week you started directly to work at the ETC?

R: Yeah, the last day of the training we went to the ETC.

I: So that was on Friday or Thursday?

R: It was on Friday I think so. We went to the ETC. All of the things they taught us here. Mr. J. first moved there to see the practical aspect of it. And for that day we were there the rest of the day.

I: So, there were no patients yet?

R: There were no patients.

I: And do you know when the first patients arrived there?

R: The first patient, if I don’t lie, arrived on the **8^th^ of September**.

I: 8^th^ of September, was that the next day?

R: It was not the next because the whole thing started on the **28^th^ of August when we started the training**.

I: Which day?

R: August 28 2014.

I: So you had a training for one week?

R: Yeah and August has 31 days.

I: Yeah. 28, 29, 30, 31, 1, 2, So the **3^rd^ of September is one week later.**

**R: Then we received the patient**.

I: Then you received the first patient? Exactly 7 days later. So, you finished the training on Friday? And then the weekend and then on Monday the first patient came. Only one?

R: Friday. No, two. **Two patients**.

I: Ok, and how did you become the supervisor?

R: I was not chosen from the beginning it was due to my hard work and the way I devoted myself. I was first chosen as a facilitator. After our own training, I and another person were asked to train a new badge.

I: Did you train once?

R: After they trained us and we went to the centre and we started receiving patients, 10 to 15. We decided to add the number, they decided to add a number. Of more staff. And when they call upon they chose us to train another [batch of] workers.

I: Ok, and did you do it only once? Did you give refreshment [courses].

R: Yes, we did refreshments. But this one we did was not a refreshment. After that training continued.

I: How many times did they hire new staff?

R: About two times.

I: So, at the beginning and then [one more time]

R: No, three times, three times. We came in, the second badge we train them. The third badge we trained them. All these numbers were increased due to the total number of patients. The more the patients, the more they added more people to work for the patients.

I: And then, as a supervisor, did you work as a sprayer or mainly overviewing?

R: Being a supervisor does not mean that you are just supervising or just spraying. I was having the knowledge of all the other areas. Knowledge in undressing, dressing and hygienists. The knowledge was all mine now, so I was supervising the whole [set of] activities within the ETC.

I: So, you was never a sprayer yourself?

R: I was a sprayer, because I was trained as a sprayer. But since I was chosen as a supervisor and I did have knowledge of all the other areas. Are you getting me? It doesn’t mean because I was a sprayer, I was not knowledgeable in all the other areas. I can even serve you as a dresser, since you [are] working in the ETC, you must be knowledgeable in all the other areas. But there was an area of specialization. Because the person that trained us said that it was necessary to set up it. You understand.

I: I understand. During the day, did you spray yourself?

R: During the day I was a sprayer myself. If I was looking the way how it was going, I do go inside about three times. I mean I look at the way how the work is. And being a supervisor, you are not just a supervisor outside. Because the main area where you are spraying is in the high-risk. Because that most matters.

I: How many sprayers were with you in one shift?

R: In my own shift we were about 4 in number. When we started the number increased till 8.

I: So, if you were with 4 people, how were the tasks divided.

R: So, one person was always staying at the gate before entering. If we are going for rounds, because we are having suspect, probable and confirmed, we were going with two people.

I: With two people?

R: No, two people for the undressing area. One person is going to be as a sprayer to be stand-by for any job inside.
I: In the high-risk?

R: In the high-risk. And if we were going for rounds now, the sprayer that is at the gate, can be changed to even a dresser. A dresser can even move to the gate to spray. You understand. Because at the gate is just spraying, you cannot compare it with the one in the high risk. So, the person that is specialized in that field, that is the person that I used to remove to go in.

I: To rotate?

R: Yes, rotation take place. Because we get concerns, the number increased to eight per shift. And when we were by 8 right now, we were having permanent sprayers. If you were at the gate, you stayed at the gate until your shift ends. If you are a dresser, you are there until the shift ends. If you go in for rounds, you go in for rounds. That is why we were having planning. When we get there, we do our planning. Four people used to go into [the ward for] suspect cases. Four for probable. Four for confirmed.

I: But 3 times four people is already 12 people?

R: 12 people per day.

I: Yeah, ok, but not only sprayers

R: Not only sprayers. Four people were involved - hygienists and sprayers.

I: Ok, and two nurses?

R: No, not nurses - they had their own planning, they were doing it different.

I: No, but you were thinking about four in suspect, probable and confirmed. So four of them is two hygienists and two sprayers?

R: No, it [the team] is going to be 3 hygienists and 1 sprayer.

I: Ok, so that is three sprayers [in total]. If you were with eight people, where were the others?

R: One is at the gate, two were at the undressing. You understand?

I: So that is five, where are the other three?

R: They are on stand-by to get in for another job.

I: So, they were prepared for anything?

R: For any other job.

I: Like the Vehicle? What else?

R: The vehicle is calling in for assistance.

I: Yeah, maybe the burial? What else?

R: When someone that is tired that is in[side].
I: Another place?

R: Well how I normally used to do. Sprayers at the gate, at the undressing room and if the workload is to high inside. We shout come out, come out, come out. Whether the place is in the high-risk, this person goes in and finishes the job. It depends on the workload. If there are so many, if the work [is] more. 4 in the morgue, 4 in the confirmed go to the morgue, we need to move a lot of people. And since we need to move a lot of people that day we cannot overstay in the high-risk, if you get in and you see that there is time. But the supervisor can call, get out, get out, get out. Because we are very time conscious. Because with time your life will stay longer. And we have to prevent that. That if you get in and you decide to work and not to protect something and prevent something you just add it up. We cannot accept. You understand. If you are in there, no matter what job. If you are for 45 minutes there, we shout get out, get out, get out. You move out. You get undressed. You rest for a while. And the other batch get dressed and get in. You [are] coming outside to replace those in the high-risk or you rather go to the undressing tent. So, the people in the undressing tent move in the high-risk. For any other job. And it was because of the workload we were all called IPC. And if you are working in the ETC you must be knowledgeable within all the aspects. Because you might be useful. They will use you in the high-risk for the DBM, for the last moment, to take it to the burial site. You understand. That is why we give another training again. Which is called IPC. We are all in the range. If you are a sprayer, you are an IPC, Hygienist, you are an IPC. A dresser can be a sprayer. While this thing started, they were giving us a field of specialization. By the way the disease it shows its phases, first phase, second phase. So, the more the patients, the more the training they were giving us in all fields. You understand. So, you can be a cook, it’s just an example, but it doesn’t mean that if you are cook, you are only able to prepare cassava leaf. You are there to cook all types of food. So, if you are an IPC, you must be all types of IPC and you get all trainings. When we started first it was area specialization. But then they were giving all rotations. So, a dresser can move to the high-risk. Because she or he has a training of all areas. So, in actual fact, because of the work load the training was provided in all areas. So, you cannot say, I am a dresser and cannot go to the high-risk. No because you have the knowledge.

I: Ok, and what was in the spray?

R: What was in the spray? Chlorine.

I: Only chlorine?

R: Water. Fresh water and chlorine that was what we were using to spray. And we have different chlorine. We mix it in two forms. We have the 0.5%, and 0.05%. 0.05% was the yellow tab, the one that was less in terms of concentration.

I: So, you had both of the concentrations in the spray?

R: Well, we were having two in the ETC. When we talk about the sprayers, that is a container, that is called the sprayer. That was available in all ETC. The mixture which is 0.05 and 0.5 was available in all corners in the ETC. But you as a sprayer, you need to know all differences of the sprayer. Because when you perform as a sprayer, there are certain duties you need to work with both of them. And if it is fresh water, it is soap. You understand. If you go in for any job like cleaning the toilet of an Ebola patient, you must walk [work?] with the 0.5 % chlorine.

I: But do you have two cans?

R: You cannot handle two cans, you must walk with one spray.

I: Ok, but what I mean, can you draw it for me? What does the sprayer look like?

R: Do you want me to draw now?

I: It doesn’t need to be perfect.

R: Ok, that is the container.

I: Ok, you have to explain it. What is it? A rope? Ok, that is a rope, and here? And what is this? Nozzle? What is that?

R: A rope. Yeah. Nozzle. The end part of the long [hose?].

I: OK, so it is a long.

R: So, in which whatever you want to spray.

I: So, it is around your body? With the rope?

R: We take them off because those in the high risk. We cannot carry it on our body. At the beginning when were started, we were carrying it. But later we start to taking it off because it can damage your PPE. And if the PPE is taking pare [?] we start to remove the ropes. And get them off.

I: So how did you carry it from that moment?

R: There was handle.

I: There was a handle. So, you had it all the time in your hand. What was the size?

R: Like this.

I: So, until half of your upper leg. So, it was quite heavy to hold it the whole time?

R: Depends how much you fill it up.

I: Ha ha, that is very true. How much did you fill it up?

R: I used to advise, the taps are available close [to] all tents. So, don’t fill it up too much, because it can be heavy. For you to carry it is, that will be difficult. So, you can put in until it is easy for you to carry.

I: But it was not bad for your clothes if you was carrying [it] the whole time?

R: But the cloves we were using that were the hard cloves

I: They cannot easily break?

R: They cannot break.

I: Did you check them?

R: We do check them, we do check them. That is why we as supervisor we get in. And we normally change them frequently. Those them are available in stock. We do change them because they can get out without you knowing. So the laundry is a very big department. They are highly responsible for our protection. The goggles, the cloves. IF they get any wholes, they put it in the waste. And chlorine the cloves again. If you wash them again. If you were using them, you had the right to fill it up with water and to see if it is bust. If it is bust, you throw it away. You again that you put it on, you have the right to check it properly. You need to check it. And the dressers again, who are responsible to bring it to the laundry and collect them they have tried, to inform the supervisor that these cloves we need to change them. So often we change them. Once in two weeks we change them. Because we are fighting the disease to go, we are not fighting the disease to stay.

I: So after two weeks, you thrown all of them away?

R: Well some that are not good we get of. Some that are still good will stay.

I: Ah ok, let me check. Can you tell us a bit about procedure changes.

R: Well the procedures used to change sometimes. Because it was a disease we were fighting.

I: How many times?

R: To my understanding depending how many times they look at the case. The centre that was responsible for the case study. The way they look at the case, the way they look at they will change it now, because they are starting now. Before you are starting, before you are going in the dressing tent, you need to wash. There is a tissue, wipe your hands. You understand. Then you put your first glove on before you start to dress. At the beginning we were using one glove. Especially for the nurses, we were using two. Then later they decided to use three before they can use the hard glove. Because of protection. That is all we were fighting for. The system can change, the procedure can change depending on the case study. Some were saying Ebola was having 1^st^ phase, 2^nd^ phase, 3^rd^ phase. The more who were not responsible for the procedure. For whatever things were coming up, the some things we can look at it. We can look at it in this way, it can fit this stuff. That’s why we were working with SOP, standard operating procedures. For whatever there is coming up, it must [be] within it. So, you see. Procedure will change depending with the changes.

I: So, do you remember how many times they changed it? I mean, I want to know for the sprayers.

R: The procedures are maintained. So, no changes, not any.

I: How did you know that you did not spray too much?

R: If you get into the high risk, imagine there was a spray now, there is another handle. The compression, the thing to compress. You first try to do what you want to do in. depending on what you want to do in, that is the number. You as a supervisor need plan two sprayers for that particular activities. Because the sprayers are the centre for that infection exercise in the ETC. So, it was a particular area of specialization that really helps in the fight of this Ebola. Especially in the ETC. So, if I am planning as a supervisor and I was giving the information we had 3 people in probable, suspect and confirmed. I was planning now how many people I am going to send in. This body [they] need to move to the morgue. Especially for the conformed and the suspect. Before our shift ends. So, depending upon the workload, I used [a] number of sprayer[s]. I get in and do that job within the time. You are not being a hero. That was our last word in Ebola. You cannot be a master in all. You cannot be a hero in all. So, asking how do I think, imagine the job you are in. You can even get in, you cannot in. Your materials get in. Just move out, get outside when there is any open space. Even if you did not work, but the fact it means you are helping somebody, you must protect your own life, before helping others. So, you cannot get exposed if you are not to walk. So, asking a question upon, the situation upon.

I: But I mean you cannot stand [it] with a lot of chlorine around you?

R: Yeah, you cannot resist it. That is what I am saying. So, if you are feeling anything, if you think the chlorine is about to affect you, put up your hand and you go outside.

I: Yeah, but the patients are still there?

R: Somebody that is dying, that is why if you are planning to move, you always put a team leader.

I: So how do know that you don’t spray too much, because the patients are still exposed to the chlorine?

R: The patients are people we care for. If you are going in for a dbm, there is a thing that we used to work is. That is a … screen. We put it across. We talk to them, the one that can move outside. Please can you go outside, they move outside, so we are able to use the chlorine. And again, you might arrange the nozzle and you may know how to spray. You cannot get in and see people and harm there more through the method you spray. You go in and spray the urine [chlorine?] where other patients are. This is the way I think you should spray. The nuzzle is going down and compress a little bit and you hold it like this and you spray like this. Then the hygienist will come and will take it over. Systematically. You understand. So, the patient you are working for cannot get affected with that chlorine.

I: can you tell me any incident [in which] there was a mistake within the sprayers?

R: Yeah it happens really. The idea of cleaning. And the toilet of the Ebola patient. Someone is afraid and stand like this and spray just like this. So, we said that is the wrong method. Because then the patient in the other tent start to sneeze. So that is bad. Because of that we decided to design it again. If you get in again, you want to use that exercise, please you cannot [be] able to spray. There are some floods in, you cannot able to spray. You just have your rubber, chlorine, mopping. You spray a little bit on the toilet, and then you remove it. The more you spray, the more the gas gets all over the tent. There were a lot of mistakes, but as the way we were going, we started to change them.

I: You start to change them? So, you used less water, and you sprayed closer to the case.

R: not closer that much. If here is the urine, I cannot come closer and you are touching it

I: No, you spray closer?

R: Yeah, yeah. Closer a bit. But make sure the nozzle cannot touch the ground. The sprayer is always the clean person. You are always clean. So, whatever you clean, must be clean, because you are in there to help to clean. So, if you go in there to clean, you must be a clean person. So, whatever you clean, you must be comfortable to the people you clean.

I: Ok, thank you a lot, I have asked all my questions.

**S4f Interview transcript Supervisor Dead body management team**

I: Pleun Welmers

R: support staff

I: Thank you very much for coming. Maybe you can tell me how you got in contact with the ETC and the Red Cross. Via an ad, via someone or someone called you? How did it go?

R: I have been always a volunteer, I came there from the year 2000. So, it is 17 years now. I have been a volunteer with any work since the outbreak began on 25^th^ of May 2014.

I: So, the outbreak was there, and you were [working] in the Red Cross and they told you they need people?

R: Yeah, they needed people. As a community mobiliser since the outbreak [began]. And later in the ETC, when the international federation came. I was [appointed] from the 14 of September 2014. My area of [work] was dead body management.

I: Ok, so that is how you call it? Dead body management. Ok, so you were first mobiliser. And that was with the Red Cross as well?

R: Yes

I: and then the IFRC came and they asked you. They asked you to help.

R: Yeah, they asked me to help. Because I was a volunteer. Because of the outbreak.

I: and then you got a training, they knew already from the first moment that you were in the DBM-team?

R: Yes.

I: OK, and who trained you?

R: D.

I: D.? He was an expat?

R: [He]was an expat.

I: He was a nurse?

R: He was a volunteer also.

I: How long did the training take?

R: Two weeks.

I: Two weeks, that is a long time. What did you learn?

R: In the trainings there was a training centre. And then they took us to the ETC. They gave us a lot of experience.

I: So you had to do some skills?

R: A lot of skills. How to take care of the dead body. How you get infected. Patients that die and so on. About what you need to know in the ETC in the high-risk area.

I: So they told you that and that took 14 days?

R: 14 days.

I: And did they tell you a little bit about the theory? What did they tell you?

R: Yes, a lot of experience. It was an outbreak. How to protect ourselves. And if you protect yourself. So we were wearing PPE. Only for 45 minutes. After 45 minutes in the high-risk area you were not allowed to stay there anymore. You have to move out. If you move out, we have a spray room. You spray yourself, you wash you hands and you are out of the high-risk area. You take off your clothes and you are supposed to be in the low-risk area. For refreshment.

I: How long did this refreshment take?

R: 2-3 hours

I: And then you could go again?

R: Yes

I: OK, and what else did you learn more?

R: A lot of trainings. [We] learned a lot. About typhoid, cholera, etc.

I: About a lot of other diseases?

R: Diarrhoea, high fever. It might be, it might be. We did not expect other outbreak. But with any other outbreak, we know how to protect.

I: Ah, OK. And that is why it was 14 days?

R: Yes.

I: You had the training with all DBM-members or with other people as well?

R: We had four teams, Team 1, Team 2, Team 3, Team 4. And we have nurses, and security.

I: But in the training was it [also] with other people? Or only with the DBM?

R: With the hygienists. They trained us with the hygienists. It was a general training.

I: It was a general training? For all the IPC’s?

R: Yeah. So the nurses were not included with us.

I: OK, so in the whole 14 days you had the training with all the IPC’s. So, there was never a training only with the DBM team?

R: Not at all

I: Not at all? So, everybody could be with you, with the DBM team? You could all switch?

R: Yeah, yeah, yeah.

I: So, there was one day for the sprayers, one day for you, one day for the hygienists.

R: Yes, one day of all.

I: Did you get [a] refresher course?

R: Hmm, sometimes.

I: OK, and what is sometimes?

R: Sometimes, every week.

I: Every week? You had a refresher course? What did they tell you?

R: Malaria, trauma, cholera outbreak.

I: So it was every week? Did it take a long time?

R: Yes. About two months.

I: Two month of training? What do you mean?

R: Two months of training.

I: Ah. So you had two weeks of general training. And you had then two months of weekly trainings?

R: Yeah.

I: And after? Did you still get any refresher trainings?

R: Yeah, we had refresher training.

I: How many times was that? Every month?

R: Every month

I: Or every week?

R Every month

I: OK, OK, so you were the supervisor? Did you get the training together? Or did you train the others?

R: Yeah. We always got the training together.

I: So you were the supervisor. You were never carrying the bodies yourself? Or what was your role as supervisor?

R: I carried the bodies from the morgue to the cemetery.

I: So what is your role as supervisor?

R: As a supervisor I need to control my whole team. Because as a boss you [are] supposed to work with them and to control them and to encourage them and not let them be afraid [of] the body.

I: Did someone check you as supervisor?

R: Yes we had expats.

I: **That person came [and saw] how you worked?**

**R: Sometimes they came to me, and they said M., you should do [it] in this way.**

**I: Was that a mistake, an accident, or did they give you the wrong information?**

**R: No, an accident.**

**I: What kind of accident?**

**R: A lot of accidents, a lot of mistakes. Sometimes we were using the body bag. And sometimes they said you should do it like that in [with?] the body bag.**

**I: OK. Were they often checking?**

**R: Yeah, the expats.**

I: You were from the very first moment [the] supervisor?

R: Up to six months

I: So you were for six months supervisor?

R: Yeah

I: What happened then? Why were you not a supervisor anymore?

R: They changed it

I: They had another supervisor?

R: Yeah

I: But you were still in the DBM team?

R: Yeah

I: And was it different then with the other supervisor?

R: No. We came together, sharing differences

I: No difference? OK. Maybe you can tell me a bit how it worked if someone passed away?

R: We had to carry the dead body

I: Let’s start first. What were you doing when there was no dead body?

R: We were sitting down.

I: You were sitting down?

R: Yeah. Expats had corridors. And then refreshment how to do.

I: OK, and sometimes you got a call that someone passed away? How did it work? You were in the low-risk?

R: Sometimes we were in the low-risk area. Every hour we went to the high-risk area. Every hour, 4-5 [of us] can wear PPE.

I: So you were already prepared with your PPE?

R: We were always prepared. In the low-risk we were waiting. At any hours

I: In your PPE?

R: No, no, no, no, no. When we were doing the job we were wearing the scrubs. When there was a call we wear PPE; we went to the dressing room.

I: That was when you got the call? You went immediately to the high-risk area?

R: High-risk area. We have triage, we have low risk and high risk, we have confirmed [cases], that were actually the patients.

I: And then you went in the PPE to the high-risk area? You went to the dead person? Did the [staff] already do something with the dead person?

R: No, only when we were in PPE.

I: No, but the nurses, did they do already something?

R: No, no, there is a dead body over there. We went there. In tent a, b or c. We had the confirmation and we were wearing PPE.

I: OK, and what did you do? What was the first step?

R: First we had the spray. We spray.

I: You spray the dead person?

**I2:** No, you go together with the sprayers?

R: we go together with the sprayers. We spray the body.

I: With how many?

R: We were four. 2 dbm and 2 sprayers. One of the sprayers. Or get to the… body bag.

**I2:** the hygienist

I: OK, so there were two dbm, one hygienist and one sprayer?

R: Yeah.

I: So, first the sprayer came. And then?

R: We opened the body bag.

I: Which [you] brought with you?

R: Yeah

I: OK, so did you do something special with that?

R: We have a stretcher. We cannot it bring directly to the morgue. Someone needs to snap it.

I: To snap?

R: To take a photo. **[Of the dead person?]**

I: OK, who did this snap?

R: We have the psycho social [worker].

I: Is that the social worker? OK, they took the picture.

R: Yeah.

I: OK, so was the bag open?

R: We open the bag after, so they take a snapshot. We close it and [bring] it to the morgue.

I: OK, so do you know what his bag looks like? What material was it made from?

R: Mmm, it is a plastic bag

I: I think the body is only allowed to touch the inside?

R: Only the inside.

I: How could you make sure that the body would not touch the outside?

R: There was a zip.

I: OK, so it was a round zip and you could open it totally. And how many could bring this body?

R: We were two. We had a spray, so every moment we could ask to spray our hands.

I: So, every action you took the sprayer sprayed.

R: Yes.

I: So, with two people you could carry this heavy weight. Two people was enough?

R: Yes.

I: **And where were you? At the top [or] bottom?**

**R: We had two at the head and we had two at the foot side.**

**I: But that is four?**

**R: Two handles.**

I: Oh, and the stretcher did it as well go in the body bag?

R: We take the stretcher and place it in the morgue with the body bag.

I: So where were the handles?

R: The handles were here and here, one-one.

I: OK, was it at the bed or what?

R: That was the body bag, and [we] put it on the stretcher. One on the head and one on the foot.

I: Maybe we can draw it? This is the bed and pillow. This is the stretcher. (Interviewer drawn bed and stretcher).

R draws. This is the body bag. This is the stretcher.

I: When it was here (in the bed) you have to take the body. You took it from here under the shoulder and at the foot.

R: Yeah.

I: In the armpit. And in the legs. And with two people was enough? And the body bag, was it already on the stretcher?

R: Yeah, yeah, yeah. No

I: Where was the bag?

R: You can put it here on the floor. He put it here and we zip it and we take it to the stretcher.

I: OK, so if I understand it right you could roll the body in the body bag, I have worked in the health care so, I know that technique.

R: Yeah, you could roll it. Ha hah.

I: You could roll it, so you put it in the body bag and you could zip it and you put it later in the body bag. And the stretcher, did you reuse it? How many did you have of them?

R: We had four stretchers.

I: So, every time you cleaned it?

R: Yeah, every time the hygienist and sprayer cleaned it.

I: And then? Where did you go with the body in the body bag?

R: We took the body in the bag on the stretcher to the mortuary. We have a temporary mortuary.

I: You put the bag there [for how long?]?

R: 24 hours

I: Do you know what they do there at the mortuary?

R: No, no, oh, we waited for the [relatives]. The [relatives] to arrive.

I2: That is why the social workers snap them, for the [relatives].

I: So they stayed for 24 hours. Was it in the high-risk area.

R: It was not really in the high-risk area.

I: So was it in the low-risk area?

R: Not per se in the low-risk area.

I: So what area was then?

R: It was a difference with the high-risk and the mortuary. Because there was a distance between.

I: But how do you [classify] it then? As a high-risk area?

R: Yes it was a high-risk area.

I: So, you [were] there in three layers of PPE.

R: We were always in PPE when we took the body to the mortuary.

I: Did you go there within the 24 hours?

R: Sometimes we can check there?

I: OK, so you went there to check. Were you the only people that went there?

R: Yes.

I: If you brought [the body] from the room to the mortuary, did other people go with you? Like the sprayers, to spray?

R: Yeah, yeah, yeah.

I: OK. When the dead body was there you went back to the undressing room and you waited there in the low-risk until you got another call?

R: Yeah, yeah, yeah, yeah

I: OK, and then another 24 hours had passed, you went back, and how did go?

R: We dressed in PPE and went with the hygienist to the mortuary. They disinfect the body bag.

I: The hygienist, or do you mean the sprayer?

R: The sprayer.

I: Or is it the same? The hygienist [and sprayer]?

R: Yes

I: So the hygienist is the sprayer and the sprayer is the hygienist. Is it another word for it? They are just the same.

R: Yeah, yeah, yeah. They are just the same.

I: OK. And you made use of the stretcher again?

R: Yeah, we disinfected the stretcher. We used the stretcher again. We placed the body bag on the stretcher and took it out.

I: Where did the stretcher stay when you didn’t use it?

R: In the high-risk area

I: In the mortuary?

R: No, not in the mortuary.

I: And you were the person who took it?

R: Yes

I: And it was only cleaned with the sprayer?

R: We always disinfect with water and chlorine.

I: Who did it?

R: I can do it.

I: You asked [for] the water and chlorine [so that] you could have the mixture. And you cleaned it?

R: Yeah

**I: Who dug the grave?**

**R: We had the gravediggers**

**I: That was not you, that was someone else?**

**R: Someone else.**

**I: And did they wear PPE?**

**R: No, only boots. We took the body from the mortuary to the graveyard.**

**I: You did it with the stretcher? Did you use a new bag?**

**R: Yeah, No.**

**I: So, you took the body to the grave, that was outside?**

**R: Outside.**

I: Was it high-risk area?

R: No, it was low-risk area

I: **So, you went with the dead body to the low risk area? And then, did the gravediggers close the grave?**

**R: Yeah. They closed the grave.**

**I2: We were not responsible to close the grave.**

**I: They did it without PPE? Only with the rainboots?**

**R: Yeah, and with the hard gloves**

**I: They had gloves. And nothing for their face?**

**R: They had masks on their face. And sometimes they used scrubs.**

**I: Not always?**

**R: Not always**

I: What was your motivation to work for the ETC?

R: I am a volunteer, I am here to help my people.

I: Had you seen dead bodies before? Many times?

R: Yeah. Many times

I: Because of your family? Or friends

R: Family, friends, during the war, after the war.

I: Ok, you were not afraid?

R: I was not afraid

I: Were you afraid for the Ebola of the patients

R: Yeah, I was afraid at the beginning, then I was not afraid anymore.

I: How did you do it at the beginning when you were afraid?

R: In the first time I was afraid seeing the dead bodies of Ebola. I just did it after two or three weeks.

I: Were you there with the first dead?

R: Take the body from the high-risk to the mortuary.

I: With the patient that passed away, did you work there already?

R: I was there on the 14^th^ of September 2014

I: You were working there?

R: The first patient was from Freetown

I: Did people help you at the [start]?

R: We had the expatriates. They helped [show us] how to take care of the body.

I: Because at the beginning you don’t know everything? Did they ever change the procedure?

R: **No within the DBM they never changed the procedure.**

**I: Never? Always and always the same procedure?**

**R: Never. Yes, yes.**

I: And how was it for you, [if] it was not really a respectful way how you are used to bury the dead bodies? How was that for you?

R: **During Ebola no traditional [ceremonial]. With traditional [burial] we have a stick. With the ETC we have a stick. From the mortuary directly to the graveyard**

I: But did you find it difficult that it was not traditional?

R: Yeah, very difficult.

I: Were there any incident you can tell me about.

R: The only mistake was the difference with the burial during the Ebola outbreak.

I: Did you make ever a mistake in the procedure?

R: No

I: you never did something wrong, there were no incidents? You cannot tell us anything about a mistake?

R: No.

I: Do you have any story or experience that you want to share you find it is important to share?

R: It was strange.

I: But do you have stories?

R: I don’t have stories to tell.
