# Supplementary material for: Trust, and distrust, of Ebola Treatment Centers: A case-study from Sierra Leone
Source: PLoS One. 2019 Dec 2;14(12):e0224511. doi: 10.1371/journal.pone.0224511 (PMC6886773; doi:10.1371/journal.pone.0224511)
Supplement: S5 File — Statements made by randomly selected citizens. (DOCX) [file pone.0224511.s005.docx]

**S5 – FOCUS GROUP CITIZENS KENEMA**

ENUMERATOR: Francis Baigeh Johnson CASE STUDY: ETC Kenema GROUP: Randomly selected people from Kenema

DATE: 11^th^ July, 2017

TOTAL ATTENDANCE: 6

Time: 10:21 AM – 12:30 PM

| Focus Group Discussion  Random  Total number in the group: 6  Place: Pastoral Centre  Date: 11/7/2017 | Selection method  Paper ruffles were made for six sets of indicators: 1) names of the community neighbourhoods; 2) street number; 4) right or left side; 5) a chosen amount of eight houses; 6) gender. Blind draws from each set selected the house (e.g. third house, left side of fifth street in neighbourhood x) and person (man or women) to ask for. |
| --- | --- |

| **RUN NO** | **CARD NO.** | **AGE RANge** | **SEX** | **OCCUPATION** | **QUESTIONS** | **ANSWERS** |
| --- | --- | --- | --- | --- | --- | --- |
|  |  |  |  |  |  |  |
|  | Ia | 3-39 | F | Trader | What is Ebola according to you? Virus, a myth, a reason to make money? | Ebola is a virus that is easily transferable from one person to another. It has symptoms that shows that it is Ebola. |
|  | Ie | 50-59 | M | Tailor |  | In my own view it is a disease that can move from one country to another through contact with a sick person. |
|  | Id | 40-49 | F | Trader |  | Ebola is a disease that is got from human to human contact. Ebola, we are made to understand, is got from animals like monkey, bat and contact with human beings that are infected. |
|  | Ia.2 | 30-39 | F | Trader |  | Most people, especially the uneducated, were saying that it is just a story for making money. |
| 1 | Ie1 | 50-59 | M | Tailor | Do you look in a different way on health facilities after the Ebola crisis? | Yes, I now see the health facilities after the Ebola crisis as improved as compared to the period before Ebola. |
| 2 | Ib1 | 40-49 | F | Trader |  | Yes, after the Ebola crisis our health facilities are more trusted now than before. Initially there was only one ambulance for the whole township. We now have over four or five, and there has also been an improvement in the supply of drugs. This improvement was quickly realized. |
| 3 | Ia1 | 30-39 | F | Trader |  | We now thank God for the health facilities after the Ebola crisis. We used to buy gloves, syringes, and most of our medicines. But now you don’t buy these anymore. You don’t buy your medicines at the hospital anymore. That is more transparent. You buy them at the pharmacy. |
| 4 | Id1 | 40-49 | F | Gardener |  | Our medical facilities have greatly improved after the Ebola crisis, [especially] in the supply of drugs, treatment and other services. |
| 5 | Ic1 | 60-69 | F | Trader |  | For that fact that you have money you can purchase whatever drugs you want before and even after. There is no change |
| 6 | If1 | 20-29 | F | Trader |  | Health facilities have now improved greatly as compared to the period before Ebola. |
| 7 | Ib2 | 40-49 | F | Trader | How did you hear of the establishment of the ETC? | The ETC was located on the way to my village. So during a visit to my village, I came across the centre and the bike rider told me it is an ETC. |
| 8 | Ia2 | 30-39 | F | Trader |  | My friend told me about the ETC |
| 9 | Id2 | 40-49 | F | Gardener |  | We were travelling in a vehicle to my village and my fellow passenger told me that’s the ETC for our district. |
| 10 | Ie2 | 50-59 | M | Tailor |  | I heard [about] the establishment of the ETC through the radio station. |
| 11 | If2 | 20-39 | F | Trader |  | My neighbors told me about the establishment of the ETC |
| 12 | Ic2 | 60-69 | F | Trader |  | I don’t know about [any] ETC |
| 13 | Ie3 | 50-59 | M | Tailor | How did you feel about the establishment? | I was greatly happy to hear this good news simply because we now have the chance to take our sick people to the government hospital for the [safe] treatment of sicknesses other than Ebola. |
| 14 | Id3 | 40-49 | F | Gardener |  | I was very happy to hear about the establishment of the ETC. |
| 15 | Ia3 | 30-39 | F | Trader |  | It came to me as a good news to hear about the establishment of the ETC. |
| 16 | Ib3 | 40-49 | F | Trader |  | We were happy to hear the good news of the establishment of the ETC. It is good that the patients were taken separately. |
| 17 | If3 | 20-29 | F | Trader |  | We were initially afraid to visit the governmental hospital for the treatment. I had to treat my sick child myself because I was afraid to go to the hospital. So the establishment of the ETC relieved me of my worries. |
| 18 | Ie4 | 50-59 | M | Tailor | How did the image of the ETC change when expatriates came to run it? | When the expatriates came, the number of deaths of patients at the centre drastically reduced. There were a lot of improvements because the expatriates know [about Ebola]. |
| 19 | Ib4 | 40-49 | F | Trader |  | We started getting survivors and reduced death rate when the expatriates came. |
| 20 | Id4 | 40-49 | F | Gardener |  | Our confidence in overcoming the virus was boosted by the coming of the expatriates. |
| 21 | Ia4 | 30-39 | F | Trader |  | Nobody was surviving before the expatriates came. We started getting survivors after their arrival. |
| 22 | Ia5 | 30-39 | F | Trader | What image did you have at the beginning? How were patients treated? Why [was it like this]? | Our local staff were only giving blind treatment to patients because they were not grounded in treating the Ebola virus. |
| 23 | Ies | 50-59 | M | Tailor |  | The local staff were not trained in the treatment of the Ebola virus. The patients were blindly treated. |
| 24 | If4 | 20-29 | F | Trader |  | The local staff were giving treatment to the patients of which they themselves did not know the function. |
| 25 | Ib5 | 40-49 | F | Trader |  | We heard that at the initial stage there was no treatment for Ebola and the patients were not properly treated. |
| 26 | Ie6 | 50-59 | M | Tailor | Did this change over time? How? Why? | Yes, it changed over time. The right treatment was now given to the patients and people were adhering to the bye-laws. |
| 27 | Ia6 | 30-39 | F | Trader |  | Yes, it changed over time because we started obeying bye-laws since those were ways to prevent the virus. Infections were reduced. People changed their behavior. My father even kept me in quarantine for three days. I was not allowed to go to the market. |
| 28 | Ib6 | 40-49 | F | Trader |  | Yes, it changed over time by strictly adhering to the bye-laws and patients were getting the right treatment. |
| 29 | Ib7 | 40-49 | F | Trader | In what perspective do you look back to the ETC? Good, or bad? Would you prefer this than other health facilities? | The establishment of the ETC was good and the patients were well treated. [But] yes, I would choose another health facility because the ETC reminds me always of my late sister. I think it is good that it does not exist anymore. |
| 30 | Ia7 | 30-39 | F | Trader |  | I would want the ETC to be re-established, because we are hearing [about] monkey pox and other [infectious] diseases which can [that need to be] be treated at the ETC. Patients were nicely treated at the ETC. |
| 31 | If5 | 20-29 | F | Trader |  | The ETC helped us greatly, and that is why I want it to be re-established. |
| 32 | Ie7 | 50-59 | M | Tailor |  | The ETC always reminds us about the number of people we lost through the Ebola virus. Therefore I wanted it to be re-located in another area. |
| 33 | Ie8 | 50-59 | M | Tailor | What good stories did you hear of the ETC? | When we started getting survivors at the ETC, [that] was a very good story to us. |
| 34 | Id7 | 40-49 | F | Gardener |  | As a result of the good treatments that were delivered, we started getting survivors. These survivors got a lot of presents. |
| 35 | If6 | 20-29 | F | Trader |  | We heard that the patients were well treated and most them started surviving. This meant that the disease came soon to an end. |
| 36 | Ia8 | 30-39 | F | Trader |  | The news of survivors discharged at the ETC was good news. |
| 37 | Ib9 | 40-45 | F | Trader | What negative stories did you hear of the ETC? | When the death toll was very high and the sound of the ambulance [was heard] that was a very negative story. |
| 38 | Ia9 | 30-39 | F | Trader |  | We heard that they used to remove patient’s blood, [and] we didn’t know why. Also the high concentration of chlorine was killing too many patients. |
| 39 | Ie8 | 50-59 | M | Tailor | How did you hear about the first survivors? What feeling did this news give you? | I got the news about the first survivor through radio, and I was very happy to hear [it]. A survivor told his story. |
| 40 | Ia10 | 30-39 | F | Trader |  | I heard the good news from a nurse who is our neighbor. I was greatly relieved. |
| 41 | Id8 | 40-49 | F | Gardener |  | I heard the news from a radio and I was happy. |
| 42 | If7 | 20-29 | F | Trader |  | I heard the news from a radio and I was happy about it. |
| 43 | Ia11 | 30-39 | F | Trader | Do patients & survivors have another image than the patients [with experience of] another health facility? How? Why? | Those patients that survived at the ETC had no problems when discharged, as compared to those [Ebla survivors from] the government hospital who had hearing problems, speech and even some experience of pain in their joints. |
| 44 | If8 | 20-29 | F | Trader |  | The ETC survivors are very much normal as compared to the survivors from the government hospital, who are still complaining of complications |
| 45 | Id9 | 40-49 | F | Gardener |  | There were vast differences between survivors and patients at the ETC and those [from] the governmental hospital. |
| 46 | Ib10 | 40-49 | F | Trader |  | The patients from the government hospital had some complications like mental retardation, speech and hearing problems. More patients from GH shake the whole time with their head. But at the ETC, there were no such problems. It implies that the people there were better treated. |
| 47 | Ie | 50-59 | M | Tailor |  | Fewer people were cured from the government hospital |
| 48 | Ib11 | 40-49 | F | Trader | How do you look at the people that worked there? And about their payment? | They were brave people who sacrificed a lot to see that the Ebola crisis is over. They were well paid. |
| 49 | Ia12 | 30-39 | F | Trader |  | I was envious of their earnings and because they were earning too much money. |
| 50 | Ie9 | 50-59 | M | Tailor |  | The way they were treating the patients. Even when they were highly paid, they still needed bribes before they could collect the dead bodies from the houses. Otherwise you were the last one they helped. I hated them so much. |
| 51 | If9 | 20-29 | F | Trader |  | They [probably refers to burial team] were disrespecting the dead who were not buried properly, and I hated them for that reason. |
