# Supplementary material for: Trust, and distrust, of Ebola Treatment Centers: A case-study from Sierra Leone
Source: PLoS One. 2019 Dec 2;14(12):e0224511. doi: 10.1371/journal.pone.0224511 (PMC6886773; doi:10.1371/journal.pone.0224511)
Supplement: S6 File — Statements made by “Taninihun” village group in 2014. (DOCX) [file pone.0224511.s006.docx]

**S6 - PARTICIPATORY LISTENING SURVEY -** FOCUS GROUP DISCUSSION MEETING HELD IN “TANINIHUN”, LOWER BAMBARA CHIEFDOM, KENEMA DISTRICT, SIERRA LEONE, 8th December 2014 MALE ELDERS

| NO | CARD  CODE | QUESTION | AGE  RANGE | MALE ELDERS (comments) | COMMENTS |
| --- | --- | --- | --- | --- | --- |
| 1 | Aa1 | What are the infectious diseases that affect your community? | 50-59 | Aa1 - Many diseases affect our community. Last month we lost one of our relatives to chicken pox [possibly measles]. | Discussion before commencement of focus group discussion. |
| 2 | Ab1 | Do you know about PPR? | 60-69 | Ab1 - Town chief, PPR has not affected our livestock |  |
| 3 | Ac1 | What sicknesses seriously affected you in the last 12 months? | 40-49 | Ac1 - Imam: the sickness that has affected us most is diarrhea (*Agbo abalie*) |  |
| 4 | Ad1 |  | 50-59 | Ad1 - Malaria is the most common sickness in this community and affects the majority of the people. | Malaria is a serious issue, as stated by the elderly. |
| 5 | Ac2 |  | 40-49 | Ac2 - Ebola is the disease currently at hand and we are confused how people can be cured of the disease. |  |
| 6 | Ab3 |  | 50-59 | Ab3 - My son died of Ebola and I was not allowed to pass and see him. | Prevented from going to Kenema at the checkpoint. |
| 7 |  | How chickenpox affects people. |  |  |  |
| 8 | Aa2 | Did anyone die of the disease? |  | Aa2 [?] Yes – VK, m., 45 yrs. old died and was buried in the town cemetery. MJ, m., also died, but the town speaker's daughter survived |  |
| 9 | Aa3 | How was the town speaker's daughter able to survive? | 40-49 | Aa3 - Prayer and immediate hospitalization. | Measles killed a lot of people. |
| 10 | Ac3 | How was chicken pox dead body handled? | 40-49 | Ac3 - Washed by 3 people. One poured hot water and 2 rubbed soap; all 3 dressed the body. |  |
|  | Aq1 | How does Ebola affect people(symptoms) | 50-59 | Aq1-Diarrhea, tiredness, loss of appetite, sore throat, Head ache, fever, Heart ache, stomach ache and vomiting. |  |
| 12 | Aq2 | Transmission of Ebola. How many people were infected? How many died and survived? Where were they buried? |  | Aq2 - 42. 29 deaths, and survivors 13. Two buried in Komende Luyama town cemetery and the rest at Nganyahun junction, Kenema district "epi-center" | JA, f., had long-term stomach pains, and comes every year for treatment.  *["Epi-center" is local coinage for an Ebola treatment centre*] |
| 13 | ? | Mode of transmission |  | Mrs. JA married in Levuma, Yawei chiefdom, Kailahun district, but was born in Komende Luyama. She long suffered stomach pain and came for annual treatment. She came with [what she and we thought was] the same pain, but this time it was Ebola. A majority of her relatives sympathizing with her contracted the disease, when they went to greet her. |  |
|  | Aq3 | Where were the dead bodies buried? |  | Aq3 - Only 2 were buried in our own cemetery, but the rest were buried in the "epi-center" in the direction of the wind. | [*Speaker thought the wind might be responsible for the spread of the disease*.] |
| 14 | Ad2  An2  Ae2  Ag1 | Have you heard about measles [smallpox]?  [*There is regular confusion among Mende speakers between smallpox, chickenpox and measles*]  How many people died? |  | Ad2 - Yes, 1960-1980. Symptoms.  Fever, Krawkraw [itchy blisters] on the skin, redness of mouth lip and eye  An2 - sore throat and loss of appetite  Ae2 - sleeping too much, blindness  Ag1- deafness of ear.  Many people died | The imam survived the measles attack in the 1960s but says many children died |
| 15 | An3  Ak2 | Prevention and treatment of measles [*smallpox*] |  | An3 - Drinking and rubbing on your skin honey and omole [*local spirit*]  Ak2 - boiling of konso [*pigeon pea*] leaf (drink the water, grind the leaf and rub).  AI1 - Boil *jasui* and drink (herb), Ah2 - Drink *yumbuyambay*  Ap2 - drink bamboo wine dreg  Al2 - wash with black soap.  **Ag2 - the infected child is not allowed to interact with the other children. He or she is taken to the farm during the day and sleeps in separate room with mother.** |  |
| 15 | Ag3  Al3  Ap3  Ah3  Am2  Ab3  Af2  Ap4  Ac2  An3 | Have you heard about TB? Symptoms?  TREATMENT |  | Ag3 - Yes. Excessive cough without sound  AI3 - Loss of weight  Ap3 - Swollen body  Ah3 - Difficulty breathing  Am2 - Vomiting with blood  Ab3 - Loss of breath when speaking  Af2 - Take to hospital for treatment  Ap4 Separate toilet and utensils  Ac2 - cough in cup or containers and not allowed to put out [spit] cough on the floor  An3 - Joint pain | Not more than 10 people were affected |
|  | Af3  Ac4  Ap2  Aq3  Al4  Ag4  As1 | What are the diseases that affect goats? |  | **Peste de petits des ruminants (PPR)**  Af3 - Ectoparasites  Ac4 - Diarrhea  Ap2 - Mange  Aq3 - Poisonous leaf (*Njapaa [lit. goat killer]*)  AI4 - Foot rot  Ag4 - Poisonous insect  As1 - Oef [?] | Many people don’t rear goats in this village |
|  | Aa5 | Who takes care of Ebola patients in Luyama? |  | Aa5 - Older youths in the family take care of sick parent or relatives | In a family, older sons or daughters take care of the father or mother |
|  | Al4 | If an adult becomes sick who takes care? |  | AI4 The adult men and women take care of adult sick people in the village, because they will be able to counsel the sick person | This is still practiced in the town |
|  | ? | If a societal head or member becomes sick who takes care? |  | Societal people within the community |  |
|  | Ag5 | Where do they carry sick people when they are unable to get a cure in the village? |  | Ag5 - We carry them to the hospital |  |
|  | As3 | How do you carry sick people? |  | As3 - By vehicle, Honda and hammock |  |
|  | Ac5 | What will the people do if there is no money to pay for transportation? |  | AC5 - The whole village will be tasked to contribute money |  |
|  | Ad6  Ae4  Al4 | How can you stop Ebola in your community? |  | Ad6 - Washing of hands with black soap and ashes 5-6 times a day and every time we use toilet. Quarantine of infected person  Ae4 – Don’t touch anyone  AI4 - Avoid public gatherings | **Compliance with bye-laws. During the outbreak no one was allowed to leave his house to visit another person** |
|  | As4  Ac5 | Are there rules in the community on Ebola prevention? |  | As4 - **Compliance with by-laws has helped us a lot**.  Ac5 - Survivors take three month before having sexual contact with their partners (90 days) | That is why no woman is pregnant in this town [*or so it was said*]. |
|  | Af4 | How do you go about the burial process in your town? |  | Af4 - Bereaved family presents a kola nut to the chief about the death of their relative. Chief informs the sanitary officer who will give the go-ahead to bury. The religion of the person will be notified. If it’s a Muslim, the elders (men or women) will wash the body and dress it. | Elderly persons from both religions will be called to wash and dress the dead person, **not necessarily members of his family**. |
|  | Ac6  Ad7  An4  Ad8 | If an adult dies? |  | Ac6 - The adult elders of that religion will wash the body. If the person is a societal member, the societal members will wash and dress the body  Ad7 - Youths are not responsible for washing dead bodies, their main role is to dig the grave, build booths for the night vigil, take message to deceased relatives in nearby villages and to carry the corpse to the grave site and cover the grave. The women will carry water to some point near the grave site and leave it there for the grave diggers to wash their feet and hands after digging the hole  An4 - Food will be provided for all those who took part in the burial  Ad8 - if a Muslim dies his burial process is handled by the Muslim people | **Youths are not normally involved in the washing and dressing of dead bodies.**  Here the Muslims will dominate the process, but Christian will also take part |
|  | An5 | Who takes or draws the clothes from the mud by the grave? |  | An5 - The last daughter/son is the one who owns the clothes because the last person is regarded as poor and unable to buy clothes for themselves, so they are given these clothes to drive away poverty. The clothes are drawn and the person runs with it to the waterside or river. The clothes are washed and dried and he or she can then use them, but they do not wash the person in the water | This process is to provide clothes for the last person in the family because they cannot afford and will not be able to fight for any clothes left behind by the deceased |
|  | Ac6  Aq4  Ae5  Ag7  Ac8 | What is the current burial practice adopted by your town? |  | Ac6 - Nobody in this town is allowed to touch a dead body, be it Ebola or not. Dead bodies are taken care of by burial team  Ag6 - nobody is allowed to touch any person whether sick with Ebola or not. We are to call ambulance  Aq4 - We want to be trained to bury sick Ebola people because the burial team is not doing anything except to dress the corpse and kick them with their feet into the grave  Ae5 - We are not happy with the way the burial teams handle and bury our people. They sometimes kick the corpse into the grave and they are not given enough soil. They treat our people like animals. When the burial team arrives, they go into the house or room alone without any family representative, drag the body out, and swing and throw it into the vehicle with speed  Ag7 - At times if a person dies of Ebola it will take more than 3 or 4 days before the burial team arrives  Ac8 - We have realized that the chemicals that are used on these bodies are too harsh and within a month the body in the grave will burn. We are not happy about that | They were not happy with the burial team. They say they do the digging and cover the body themselves, so what is the burial team paid for. So they want to be trained in how to bury their relatives so that their relative will be buried in peace. The Elders were happy with the way the interaction [*discussion*] went on |

PARTICIPATORY LISTENING SURVEY - FOCUS GROUP DISCUSSION MEETING HELD IN “TANINHUN”, LOWER BAMBARA CHIEFDOM, KENEMA DISTRICT, SIERRA LEONE, 8th December 2014 **FEMALE ELDERS**

| No | Card  Cod You can hardly talk about n = 6 as a "sample". | Question No | Age | Elderly Women | Conclusion |
| --- | --- | --- | --- | --- | --- |
| 1 | Ba1 | Sickness |  | Ba1 - Malaria |  |
| 2 | Ba1 | What is the proof that the person has malaria? | ? | Ba1 - The child's blood was taken and result showed malaria.  White eye membrane, pale body, weakness and sweating. | Malaria was reported as a major disease in this town. It is possible that some of the high fever was not actually Ebola, but cases were rushed to Kenema [*with little feedback*] and quarantine was imposed [*making it impossible for villagers to check on their sick relatives*]. There may have been cross-infection in Kenema |
| 3 | Ba2 | When the person became infected where did they take her? |  | Ba2 - Child died in Kenema hospital and was brought back to village for burial |  |
|  | Ba3 | Burial, who does what? |  | Ba3 - Girls are washed by women and men do the burial |  |
|  | Bb1 | Signs of malaria | 40-49 | Bb1 - Fever, quietness, wash with cold water, and soon catch fever | So if they could wash the people with hand wrapped in cloth, then maybe it was not Ebola? |
|  | Bb2 | What were they told was the cause of malaria? |  | Bb2 - Mosquito | Komende Luyama is a cool, hilly location; is mosquito numerous? |
|  | Bb3 | What happened to the sick person when they took him to the hospital? |  | Bb3 - In hospital, they draw our blood and malaria was reported. Gave drugs for 3 days and he recovered |  |
|  | Bb4 | What do they do with the dead body before burial? |  | Bb4 - Wash the dead body and prepare for burial |  |
|  | Bc1 | Did you use your bare hands? | 60-69 | Bc1 - They wrap cloth on their hands and wash the body |  |
| 1 | Bc2 | What happens to the women after they have dressed the body? | 60-69 | Bc2 - The women will stay at home while the men bury the corpse | Since this is a Muslim community, the women are not allowed to go to the burial site; they will have to stay home |
| 2 | Bc3 | What other sickness have you experienced in this town? | 60-69 | Bc3 - Convulsion, has high fever |  |
| 3 | Bd1 | Is there any other sickness? | 60-69 | Bd1 - Yes, lebola [*Ebola*]. My child died and the family was quarantined, but the grandchild survived. I also saw a wife die, but the husband survived |  |
|  | Bd2 | What was the sign to show the person had Ebola?  What happen when people show such signs? |  | Bd2 - High fever and no drug works for this fever  My daughter carried a 2 month pregnancy; Ebola aborted the pregnancy and was reported to the soldiers, but my daughter died and lay in pool of blood |  |
|  | Bc4 | What happens when the sickness gets worse? |  | Bc4 - Call the ambulance to take her to hospital |  |
|  | **Bf1** | **What happens when the person is taken to the hospital?** |  | **Bf1 - There was no word from the medical people. But when the driver of the ambulance comes [*again*] the people will ask him and he will say if the person is dead, but they don’t hand over the body to the community** | **Get Ebola death information from ambulance drivers. Possibly, drivers do not give correct information** |
|  |  | What happened to the victim? |  | Child was taken to the school and quarantined |  |
|  |  | How did the town know that the person had died? | 20-25 | The ambulance came for the body and we knew that person had died. The quarantine woman who slept with the child contracted the disease and the brother who came from Yawei chiefdom contracted the disease from the sister and also died. |  |
|  | Be1 | Burial | 600-69 | Be1 - Family called to say they will give us good burial |  |
|  | Be1 | How good was good? |  | Be1 - They promised to take photos of the burial process |  |
|  |  | Prevention of Ebola |  | Observe the rules:  Do not visit another house, suspected cases are immediately quarantined, quarantined people are not allowed to share the same wells and food with unaffected people |  |
|  |  | Where did the first case of Ebola come from? |  | Yawei, Kailahun district. The woman had stomach pain. Two days later she died. The bike rider that brought her also died. |  |
|  |  | Cases of hernia? | 40-49 | ?? - Mohamed had hernia, but there was no money to take him to Kenema; he died and was buried by the burial team without washing the body |  |
|  | Bg1 | Onchocerciasis |  | Bg1 - has ulcer, [and] onchocerciasis, was hospitalized, 2 months later contracted Ebola and died | People think he contracted Ebola from the hospital |
|  |  | Burial | 60-69 | My brother died, was washed with gloves, wrapped in *kasanke* [shroud] and buried, but women were prevented from going to the burial site. Woman and man when they married are not separated even during death, but the woman was not allowed to witness the burial of the husband. The elder male child took the body and washed it |  |
|  |  | Why was the woman prevented from sitting on the mat after the death of the husband? |  | Because of Ebola, but the woman put on a charm and wore a black and white cloth for 40 days and washed at the fringe of the river or swamp (*kpete*) | People are not allowed to touch and visit each other at home, so [*even if she is put*] on the mat no-one can visit |
|  | Be3 | What happens to the last child when the parents die? | ?? | Be3 - The cotton cloth or lappa is slightly put under the grave mud, it is drawn with some mud and placed on the head of the last born. He will run to the river and the cloth is washed in the water and hung in the house to dry. The adults will wash themselves |  |
|  | **Be4** | **What happen when the body is taken to the grave?** |  | **Be4 - One person will enter the grave to receive the body sent down by two people. They will ask all present if deceased has to pay anyone? If someone answers, the family will pay that person the amount owed. This must be settled before the person is buried** | **Debts have to be repaid. The person entering the grave will have to cover the deceased with stick and cover these with leaves before mud is placed on the leaves. This will prevent mud touching the corpse** |
|  | Bh1 | Who cares for the wife or husband when one of them dies? | 40-49 | Bh1 - It is either the husband or wife, but in both cases the children or relatives will be responsible. Any member of the family recognized by relatives or the town |  |
|  | Be5 | Where are sick cases mainly referred to? | 70-79 | Be5 - To Kenema, by the nurse and they will have to pay for their own transportation. If there is no money, they will borrow from friends |  |
|  | Be6 | How is the first born buried? |  | Be6 - The body is laid on a leaf called *pomamagbe* [*Newbouldia laevis*] and buried with rags. Men will dig the grave; men bury boys while girls are buried by women.  The mother of the first born will put dirt from the dustbin on the child in the grave |  |
|  | Be7 | What happens to an adult first born at death? |  | Be7 - He is called *Gboglui* and is buried the same way as the boy |  |
|  | Bl1 | How many times do wife and husband sit on the mat? |  | Bl1 - Once |  |

PARTICIPATORY LISTENING SURVEY - FOCUS GROUP DISCUSSION MEETING HELD IN “TANINHUN”, LOWER BAMBARA CHIEFDOM, KENEMA DISTRICT, SIERRA LEONE, 8th December 2014 **YOUTH (M and F)**

| NO |  | Question | Age | Youth | Conclusion |
| --- | --- | --- | --- | --- | --- |
| 1 | Ca1 | Sicknesses you have experienced in this town during this past one year? | 10-19 | Ca1 - Malaria |  |
| 2 | Cj1  Ca2 | What are the signs of the sicknesses you have mentioned? | 20-29  10-19 | Cj1 - Constipation, blockage of nose, difficulty in breathing.  Ca2 - Headache, body and joint pain and cold, dizziness |  |
| 3 | Cj2 | Causes of these sicknesses? | 20-29 | Cj2 - The type of food we eat, carry heavy load on head and don’t eat fruits |  |
| 4 | Cj3 | Have these sicknesses affected any one? | 20-29 | Cj3 - Plenty of us |  |
| 5 | Ca3  Cd1  Cb1 | What treatment do you take? | 10-19 | Ca3 - Panadol, chloroquine, pain killer  Cd1 - Sudrex, cold cap  Cb1 - Native medicine such as njasue, yumbuyambay, gbangba and sometimes buy medicine from "pepper doctor" [*itinerant quacks*]. |  |
| 6 | Cd2  Cd3 | Has the sickness killed anyone in the village? |  | Cd2 - No one has died of it  Cd3 - Yes, my grandmother name HA, aged 60. |  |
| 7 | Cg1 | How did you hear about Ebola? | 20-29 | Cg1 - Radio |  |
| 8 | Cp1 | How do people contract this disease? | 20-29 | Cp1 - eating animals that have the Ebola virus and those in the bush  Cl2 - eating animals in the bush and touching people infected with the virus | But that was not the case in Luyama |
| 9 | Cz1  Cg2 | Describe the symptoms of Ebola | 20-29 | Cz1 - Cold, headache, pain  Cg2 - Joint pain and pain in every part of the body |  |
| **10** | **Cz2** | **Has anyone been affected?** | **??** | **Cz2 -Yes, more than 30 people were infected** |  |
| **11** | **Cg3** | **How did the other people come in contact with infected person?** | **20-29** | **Cg3 - visited patient in hospital and those who visited contracted the disease** | **Sympathized with woman long-standing stomach pain** |
| 12 | Cl1  Cq1 | What happen after hospitalization? | ?? | **Cl1, Cm1 - The infected woman died in the village hospital**  **Cq1 - was buried in the village commentary and all those who came into contact with her died.** | **Clinic in the town** |
| 13 | **Cq2**  **Cg4** | **What did Government do after the death of this woman?** | **??**  **20-29** | **Cq2 - Quarantined the people who came into contract with the sick women; 42 were sick and 29 died, but 13 survived.**  **Cg4 - See list of people who died, below** | **Soldiers came to town, quarantine lifted 4 days ago (4/12/14)** |
| 14 | Cr1 | How do you treat people infected with this disease? |  | Cr1 - Take person to health center for treatment |  |
| 15 | Cj4 | Who took care of the sick person? | 20-29 | Cj4 - a 40 year old sister |  |
| 16 | **Cg4** | **Other sicknesses that affect us in this village?** | **20-29** | **Cg4 - Hernia affects most of us youth** |  |
| 17 | **Cu1** | **Symptoms of hernia?** | **??** | **Cu1 - Stomachache, movement in the stomach like worm, cold, pain in groin (*kpewie*), vomiting and nausea (*ndemalei*)** |  |
| 18 | **Cu2** | **Causes of this sickness?** |  | **Cu2 - Carrying heavy loads, hard work and walking long distances** |  |
| 19 | Cl3 | Treatment? |  | CI3 - take paracetamol, and take person to health center for treatment |  |
| 20 | **Cl4** | **Did anyone died of the sickness?** |  | **CI4 - Yes, 2 men, MM, age 25, and MK age 40. They were taken to the hospital but it was too late, and they died** |  |
| 21 | ? | Who took care of them? |  | MM was taken care of by his mother and MK by his sister; both were buried in the normal way | Role of close relatives as carers |
| 22 | Cb2 | Do you know about PPR? | 20-29 | Cb2 - Yes |  |
|  | Cb3 | Signs and symptoms of PPR | 20-29 | Cb3 - when animal eats an unwanted plant | Don’t know the disease |
| 23 | Cb4 | How do you prevent it? | 20-29 | Cb4 - Take the animal to the stream and wash it, then rub it with blue. |  |
| 24 | Cf1 | Have you heard about smallpox? | 20-29 | Cf1 - yes, it’s a skin infection |  |
| 25 | Cf2 | Symptoms? | 20-29 | Cf2 - Fever, rash with water under it, mouth becomes red and cold sets in |  |
| 26 | Cf3 | Causes of the disease? | 20-29 | Cf3 – we do not know |  |
| 27 | Cf4 | How do you prevent it? | 20-29 | Cf4 - isolation from none infected people |  |
| 28 | Cf5 | Treatment | 20-29 | Cf5 - we grind Konso bean [*pigeon pea*] leaf and rub on the skin. If no improvement, we take the person to hospital |  |
| 29 | Cf6 | Did any person die of the sickness? |  | Cf6 - no |  |
