# Supplementary material for: Trust, and distrust, of Ebola Treatment Centers: A case-study from Sierra Leone
Source: PLoS One. 2019 Dec 2;14(12):e0224511. doi: 10.1371/journal.pone.0224511 (PMC6886773; doi:10.1371/journal.pone.0224511)
Supplement: S7 File — (DOCX) [file pone.0224511.s007.docx]

**S7**

**EBOLA LISTENING SURVEY PROTOCOL**

**DFID Project**

**Guide for focus group meetings [rev. PR 1st December 2014]**

**Paul Richards – study director**

**Esther Mokuwa – field team leader**

**OBJECTIVE**

The current outbreak of Ebola Virus Disease (EVD) is the largest ever recorded. Evidence suggests spread has been almost exclusively through human-to-human contact. Very little is known about the social factors that drive the spread of the disease and the ways in which it might be stopped. In part, this is due to the limited research in the Ebola crisis asking people their perceptions. We propose to implement a pilot study in 25 villages in Sierra Leone where over the years as a research team have built good relations.

We are interested to learn how people understand Ebola infection risks and what they think they might do to protect themselves. Key questions "are there any infectious diseases and or sicknesses that have been experienced in this community in the past 12 months?", and "what steps, if any, have individuals or the whole community taken to limit the spread of these infectious diseases or sicknesses?"

Note: We do not convey health messages but will take down any questions the community has for the authorities. These questions should be formulated as clearly as possible and we will undertake to deliver them to the MoHS and send confirmation that they have been received. We will do our very best to get community questions answered by competent authorities.

**FIELD PROTOCOL**

***a)*** ***Pre-Survey Activities***

**Informing Paramount Chiefs and Section Chiefs**

Before contact is made with villages, *LETTERS OF INTENTION* are to be sent to the appropriate Paramount Chiefs to alert them to our activities. These letters are notification of the intention to undertake information-gathering activity. Appropriate contact information is included in the *LETTER OF INTENTION* in order for any questions or concerns held by the chiefs to be addressed.

**Invitation to Participation**

In advance of survey activities, runners are to be sent to each village with a *LETTER OF INTENTION* to be delivered to the chief. The runner is required to review the letter in detail with the chief, explaining the proposed date of arrival, number of enumerators, and a summary of activities proposed. Permission is requested by the chief for activities to be undertaken. If permission is denied, the runner seeks to determine whether an alternative date is available. If the chief refuses the research team to visit at all, the runner informs the project leader immediately and a new village is selected from the same geographical area. Scheduled activities are undertaken only with the chief’s permission.

***b) Schedule of in-village activities***

After arrival in the village, the team leader will present the team and its purpose to the Town Chief. After securing his approval to carry out the program, the schedule of activities is as follows:

1. Meet the village
2. Undertake focus group activity
3. Thanks, and invitation for follow-up activities
4. **Village Meeting**

A village meeting is called by the Town Chief, or by another village authority. Give enough time for village members to be informed and brought together so that a majority of villagers is present. Introductions of important village members are made. The enumeration team members introduce themselves and their purpose in the village. Give a brief summary of activities to be undertake and what is expected of participants.

1. **Focus group activity**

**Group selection**

We will work with THREE groups – adult men, adult women and youth (THIS LAST GROUP TO INCLUDE SOME CHILDREN, IF POSSIBLE). Keep records of THE SOCIAL statuses of participants ("big people" [*ta gbakoi*], commoners, strangers). Use the "speaking card" system to track who contributes and how many times. Allow discussion to flow (use prompts only when conversation falters, or if it is clear that points in prompt list have not been mentioned). Allow a range of answers to be given. [Issues where a range of answers is given can be ranked.]

**PREAMBLE TO FOCUS GROUPS**

When a remark is made the second facilitator (who acts as group secretary and recorder) marks the relevant three element code on a card given to the speaker. The first letter (upper case) indicates which of the three focus groups per village the speaker is speaking in. The second (lower case letter) indicates the order in which the speaker joined the conversation. The third element – a number - refers to the order in which the speaker made his or her remarks. “Aa1” attached to a record of a statement indicates thus that the speaker participated in the focus group for elder males, was the first speaker in that group, and that this was the first of several remarks made by that If a person wants to speak more times the RAs will make and give out additional card(s). The cards are then used to track which different persons speak, about what topics, and in what order, but make it clear to participants that no names or other PERSONAL ID will be taken. The cards will provide an ANONYMOUS TRANSACTIONAL RECORD of the various conversations.

**TO THE TOWN CHIEF**

- Visit Town Chief and offer the conventional "gift" to state your business
- Introduce yourselves to the chief as a research team (members PLUS SUPERVISOR) from Njala University
- Ask the chief to call a short village meeting.

**IN THE VILLAGE MEETING**

- Offer prayers and state business
- Ask community to introduce elders of the community
- Give another token (*famaloi*) in public through an elder to the chief and respond to the conventional question “why has *famaloi* been given?”
- Explain that the purpose of meeting is to ask about infectious diseases or sicknesses.

**PROMPTS (STARTER QUESTIONS)**

- What outbreaks of infectious diseases or sicknesses have people experienced in the village?

[If these are numerous limit discussion to recent years; if initial answer is "none" extend the period backwards to historical epidemics, such as smallpox].

YOU SHOULD ALLOW AN INITIAL DISCUSSION ABOUT 'INFECTIOUS DISEASE' AND MAKE NOTES ON ANYTHING SPECIFIC TO LOCAL CONCEPTIONS. Get people to list examples of infectious diseases. Also, as a check, ask whether they consider malaria to be infectious (and if so, in what way).

***NOTE***: People may ask you to give an example of the sort of diseases or sickness you are interested in. You will not answer directly but say "any diseases or sickness you want to mention". If people still want to clarify DO NOT OFFER EXAMPLES OF YOUR OWN, but THEY can offer examples and you can give a response, e.g. "yes, that sort of disease or sickness". Explain you know little or nothing about disease or the area and are interested only to hear from them. Take careful note of and report any ways villagers might try to "fish" from you some assumed hidden purpose of the meeting. Explain you have no medical background, nor do you work for any ministry. You are simply collecting data, like in a census.

**A. FORMATION OF FOCUS GROUPS**

Ask the community to divide themselves into three groups [self-sorting]:

1. Male Elders
2. Female Elders
3. Youth (male and female)

[Children - see note below]

RAs should try and ensure that the groups "sort" appropriately, but let the community decide in dubious cases (whether the Youth Leader is also an Elder, for example). Write down in your field notebook how this sorting is done by the community.

**Children**: we are keen to hear from children. Please assess during training. And during the field pilot, whether it will be possible to manage a FOURTH group, of children. **Care-givers must give permission**.

**B. FOCUS GROUP ACTIVITY**

- Each group should have two facilitators; the second one has responsibility to act as recorder and to manage the “speaking card” system.
- In each group the lead facilitator will explain in detail the purpose of the meeting, and the methods of answering/responding to questions.
- Ask the group how the meeting should be run smoothly, to allow everybody to have opportunity to speak.
- A pile of cards of an appropriate colour will be assigned to the group (Green, Pink and Yellow, for male elders, female elders and youth respectively).
- Each person in the group should be labeled A, B, C. (etc., as the case may be).
- Each card should be numbered 1, 2, 3 (and more, if needed).
- Each group facilitator should prepare the cards ("speaking cards") as follows:
  - - Aa1, Aa2, Aa3, etc. – (first respondent)
    - Ab1, Ab2, Ab3, etc – (second respondent)
    - Ac1, Ac2, Ac3, etc – (third respondent)
- Explain that each card is a permit to speak

***NOTE:*** - in the event a respondent wants to talk more than three times the second facilitator prepares additional cards of the correct colour, labeled in sequence (thus Aa4, Aa5, etc.)

1. The first person to raise his/her hand to talk iss given a “speaking card” and when finished on that topic hands in their first card (Aa1 thus signifies as follows: A = Elders group; a = first respondent in elders’ group; 1 = first card). For the same person talking for a second time a second card (Aa2) is used (Aa2 = A group; a = first respondent; 2= second card), etc.
2. The facilitator collects each card as it is "played" and notes down against the card reference letter and number the topic raised.
3. This will allow the actual pattern of commentary by informants and order and frequency of topics to be reconstructed without breaching the anonymity of the speaker.
4. Facilitator should make a list of all diseases or infection sources as they are mentioned by the group. Encourage discussants to switch among these topics. If groups seem to get stuck on one topic alone prompt a shift by asking "but what about X [or Y, where X and Y are disease topics already listed]?”
5. Enumerators then summarize the discussion with the group.
6. In conclusion, participants in the groups should be thanked. It should be stressed that no responses will be identified by names.

***PLENARY*: The three groups will then come together for a concluding session. Lead enumerator will then be permitted to facilitate a general discussion on all groups' work.**

The lead enumerator reads out the all gathered discussions for logistical and clear information on diseases and sicknesses. The group discusses any discrepancies.

**C. FOCUS GROUP PROMPTS**

**[NOTE - these are "prompts". Use them only if the focus group turns in that direction but seems to need help. Do not use them as a list of set questions that must be asked]**

**1. What serious sicknesses have been experienced in this village over the**

**previous 12 months?**

[Use local names or descriptors for diseases, do NOT prompt for Ebola]

1a Further prompt (after diseases have been listed): did anybody die from these sicknesses (ask for each in turn).

- Names and ages of the people who died.

- Ask where they were buried (in this village, or other location

[get name of place, and chiefdom]).

[Note any relevant comments about whether these deaths were considered unusual - e.g. caused by witchcraft, affected mainly one group, such as strangers, women or "big people"]

**2. Questions about epidemics (smallpox, TB, PPM [goats]).**

2.a Further prompts on smallpox

- Do people have any memories of smallpox?

[get description of symptoms]

- if yes, how many people were affected

- how did people prevent spread of infection?

[Note any evidence relating to quarantine]

2.b Further prompts for TB

-Do people have any memories of TB [tuberculosis]?

[get description of symptoms]

- if yes, how many people were affected

- how did people prevent spread of infection?

[Note any evidence relating to quarantine]

2.c Further prompts for PPM [ditto] (ROLAND: how do people identify this?)

- Do people have any memories of PPM?

[get description of symptoms]

- if yes, how many goats were affected

- how did people prevent spread of infection?

[Note any evidence relating to quarantine]

1. **Who cares for seriously sick people?**

3a Further prompts

- if an adult man becomes sick who will care for him?

(specify the relationships of people likely to be involved)

- if an adult woman becomes sick who will care for her?

(specify the relationships of people likely to be involved)

- if the man or woman has no husband or wife who will care for him/her?

(specify the relationships of people likely to be involved)

1. **If the sickness cannot be cared for in the village describe what arrangements are needed to send the sick person to a hospital or health centre in another location?**

4.a Further prompts

- how will the person be transported, and what will it cost?

[specify target health facility and mode of transport]

- who will decide? (collect a range of typical instances)

[sick person, husband/wife, others in family [specify]?

- what happens if there is no money to pay transport or treatment fees?

**5. Have people heard of Ebola sickness? [Yes/no?]**

**[note if there have been any actual cases in this village]**

5.a Further prompts (if yes to question on whether they have heard of Ebola)

- ask for a description of the disease and its causes

- how does it spread?

- how can people protect themselves from catching Ebola

**6. Ask factual questions about preparation of bodies for burial and actual burial.**

6.a Further prompts: how are bodies prepared for burial?

- Washing (get a description of the process)

[who does what, and how are they related to the deceased?]

- Note any uses of water collected or mud created during washing

- How and with what are bodies wrapped for burial?

6.b Further prompts: how is burial undertaken

- Who carries the corpse? (names, ages, relationship to deceased, if any)

- For burials in the previous 6 months where were people buried

[in this village or elsewhere?]

- if elsewhere why, and how was the body transported?

- who was involved in transporting the body?

**GENERAL COMMENT**

If conversation turns spontaneously towards these topics (and ONLY IF)

- collect any description from older people about smallpox epidemics (e.g. if the village ever tried to quarantine people)

- ask for a description of what a man or woman MUST do if the husband or wife dies

- ask for a description of what the parents MUST do if a first child dies.

[**SUGGESTION**: be alert to any suggestions regarding personal protection and quarantine; pass no opinion on these issues but record every suggestion or comment in as much detail as possible, and where appropriate ask for further elaboration. If quarantine is mentioned [e.g. for smallpox] probe HOW this was organised, where people were quarantined [at home, in the farm-house, in the bush], and how care was organized.

**THANKS, and answer any questions on follow-up activities**

**Does and dont’s**

Field Team all to follow training protocol on reducing risks of Ebola infection [guidelines from GOAL/RESTLESS DEVELOPMENT]

- Only work in villages where we have worked before (ABC, Gola survey, etc)
- Do not go to villages with current known Ebola cases
- Bring own water and food
- No overnight stay in village (distant villages - carry a tent and use it if a session overruns or can only be arranged in the evening).

**Informed consent RAs**

Create form – example below

**Informed consent for villages**

Create form – example below
