# Supplementary material for: Trust, and distrust, of Ebola Treatment Centers: A case-study from Sierra Leone
Source: PLoS One. 2019 Dec 2;14(12):e0224511. doi: 10.1371/journal.pone.0224511 (PMC6886773; doi:10.1371/journal.pone.0224511)
Supplement: Attachment — Submitted filename: PLOS ONE rebuttal letter.docx [file pone.0224511.s008.docx]

PLOS ONE

Editorial Team

Dear editors,

Many thanks for soliciting reviewers for our piece and for their and your useful comments. We have revised the manuscript thoroughly and have done the following to address the requests:

General revisions requested by the editors and reviewers

1. Information on methodology: we have now included much more detail on our methodology: information on the wider study this manuscript emerged from; a clearly formulated research question; number of potential participants approached; training of the interviewers; a table detailing the status groups and numbers of research participants; information on sampling recording; transcription and analysis (as requested by the editors and reviewer 1)
2. Interview guides and data files: we have now included focus group guides, as well as the data from focus groups and from key-informants interview transcripts as supporting information. The questions asked in focus groups are listed in a column on each sheet of the focus group transcripts.
3. Ethics statement: we have amended the ethics statement to confirm that the Sierra Leone Ethics and Scientific Review Committee has approved this study.

Specific issues raised by reviewer 1:

1. “Third, the statement on line 113 (“The approach was largely qualitative”) raises questions – are quantitative data available? If so, will these data be used in the current study? Why or why not?”

The bigger overarching study on trust in medicine after the Ebola epidemic in Sierra Leone, Ghana and Uganda largely utilized qualitative methods, but included a smaller quantitative survey in all three countries. The data analysed here, however, was collected in a different study period and with a slightly different thematic focus. In this manuscript we do not draw on the quantitative material, as it is not relevant to the topic of this manuscript.

2. “Getting a group of survivors to talk in a focus group was a challenge.” – but do not follow up. Why was this difficult and how did researchers approach this challenge? What are the implications of this challenge for the validity of the results?

We provide some additional information on this question in lines 367 – 373 of the manuscript.

3. “In general, in the Results section, I am confused about which results are derived from focus groups and which are derived from interviews. Please provide some additional structure to this discuss to clarify the sources of data being discussed.”

The data in this paper are from focus group interviews and key informant interviews undertaken in 2017. All this material is now provided in the file of supporting material. Additionally we now explain that one of the focus group interviews in 2017 was conducted among villagers in a community (“Taninihun”) first studied at the height of the Ebola outbreak in 2014. We include in the supporting material questionnaire interview and focus group data from this earlier study as a “baseline” for the current analysis. Comparisons between the two data sets (for 2014 and 2017) are briefly discussed. But the paper primarily analyses the focus group and key-informant interview materials from 2017. Given the clarifications now added, we do not see a necessity to re-structure our results or discussion section.

4. “This sentence particularly stands out to me: “In short, restoring trust in medicine requires Ebola Virus Disease to be re-contextualized within a broader framework of concern for the health of all citizens.” In my opinion, this issue is actually the backdrop for the entire study and should be emphasized more strongly”

Thank you for this point, we have emphasized it more throughout the manuscript.

Specific points raised by reviewer 2

1. “Thus, the restoration of the confidence in biomedicine called for by the authors is illusory if it is only mentioned during Ebola. In times of crisis, it appears very late to lay the foundations for a relationship of trust with biomedicine (or to strengthen a health system that has been abandoned since the advent of structural adjustment policies...). Preparedness must also be considered at the level of ordinary health systems!”

We thank the reviewer for this point and his clear phrasing of it. We have included it duly credited in the manuscript.

2. Lay workers: underestimated informal communication actors

We completely agree with this point. We have now included a table that details that our study included non-medical personnel at the ETUs, in particular dressers, cooks and social workers.

3. The ambivalent figure of the Ebola survivor

We completely agree with reviewer 2 on this point, but we believe that expanding our discussion on the ambivalence and stigma faced by survivors would distract from our core message. We agree that more research on survivors and their experiences is much needed, and we welcome the work done in the piece by Gomez-Temesio that reviewer 2 refers to. However, since this is not the main focus of our paper we have chosen to not expand on this point here.

We would like to sincerely thank the reviewers and editors for their close and constructive engagement with our manuscript. We are convinced the revisions have helped the clarity and quality of the paper, and hope you find our revisions satisfactory.

Kind regards,
